# Blockage of glycolysis by targeting PFKFB3 suppresses the development of infantile hemangioma

**DOI:** 10.1186/s12967-023-03932-y

**Published:** 2023-02-06

**Authors:** Kaiying Yang, Tong Qiu, Jiangyuan Zhou, Xue Gong, Xuepeng Zhang, Yuru Lan, Zixin Zhang, Yi Ji

**Affiliations:** 1 Division of Oncology, Department of Pediatric Surgery, West China Hospital of Sichuan University, #37 Guo-Xue-Xiang, Chengdu, 610041 Sichuan China; 2 Department of Pediatric Surgery, Guangzhou Women and Children’s Medical Centre, National Children’s Medical Centre for South Central Region, Guangzhou Medical University, Guangzhou, 510623 China

**Keywords:** Infantile hemangioma, Glycolysis, 6-Phosphofructo-2-kinase/fructose-2,6-bisphosphatase 3, Angiogenesis

## Abstract

**Background:**

Infantile hemangioma (IH) is the most common tumor among infants, but the exact pathogenesis of IH is largely unknown. Our previous study revealed that glucose metabolism may play an important role in the pathogenesis of IH and that the inhibition of the glycolytic key enzyme phosphofructokinase-1 suppresses angiogenesis in IH. 6-Phosphofructo-2-kinase/fructose-2,6-biphosphatase 3 (PFKFB3) is a metabolic enzyme that converts fructose-6-bisphosphate to fructose-2,6-bisphosphate (F-2,6-BP), which is the most potent allosteric activator of the rate-limiting enzyme phosphofructokinase-1. This study was performed to explore the role of PFKFB3 in IH.

**Methods:**

Microarray analysis was performed to screen the differentially expressed genes (DEGs) between proliferating and involuting IH tissues. PFKFB3 expression was examined by western blot and immunohistochemistry analyses. Cell migration, apoptosis and tube formation were analyzed. Metabolic analyses were performed to investigate the effect of PFKFB3 inhibition by PFK15. Mouse models were established to examine the effect of PFKFB3 inhibition in vivo.

**Results:**

PFKFB3 was identified as one of the most significant DEGs and was more highly expressed in proliferating IH tissues and hemangioma-derived endothelial cells (HemECs) than in involuting IH tissues and human umbilical vein endothelial cells, respectively. PFKFB3 inhibition by PFK15 suppressed HemEC glucose metabolism mainly by affecting glycolytic metabolite metabolism and decreasing the glycolytic flux. Moreover, PFK15 inhibited HemEC angiogenesis and migration and induced apoptosis via activation of the apoptosis pathway. Treatment with the combination of PFK15 with propranolol had a synergistic inhibitory effect on HemECs. Moreover, PFKFB3 knockdown markedly suppressed HemEC angiogenesis. Mechanistically, inhibition of PFKFB3 suppressed the PI3K-Akt signaling pathway and induced apoptotic cell death. More importantly, the suppression of PFKFB3 by PFK15 or shPFKFB3 led to markedly reduced tumor growth in vivo.

**Conclusions:**

Our findings suggest that PFKFB3 inhibition can suppress IH angiogenesis and induce apoptosis. Thus, targeting PFKFB3 may be a novel therapeutic strategy for IH.

**Supplementary Information:**

The online version contains supplementary material available at 10.1186/s12967-023-03932-y.

## Background

Infantile hemangioma (IH) is the most common benign tumor in infants, with a prevalence of 4–5% [1]. IH has a characteristic growth pattern: it usually proliferates during the first year of life, and this stage is followed by a slow spontaneous involution lasting for several years [2]. It is widely accepted that angiogenesis plays a vital role in the pathogenesis of IH [3, 4]. Since 2008, when it was serendipitously discovered to effectively treat severe IH, propranolol has been the first-line treatment for IH, and this drug acts by targeting angiogenesis [1, 5–7]. Several critical signaling pathways regulating angiogenesis, including the ‘vascular endothelial growth factor (VEGF) and VEGF receptors pathway’, ‘PI3K/Akt/mTOR pathway’ and ‘HIF-α-mediated pathway’, have been demonstrated to participate in the development of IH [3, 4]. However, the exact pathogenesis of IH has not been fully elucidated.

Sprouting angiogenesis is characterized by a transition of the endothelial cell (EC) phenotype from a quiescent state to a highly active, proliferative and migratory state under stimulation with proangiogenic growth factors, particularly VEGFA [8]. Therefore, a large amount of energy is required to support EC proliferation and migration during angiogenesis. Recently, the metabolism of ECs, especially glycolytic metabolism, has been recognized as a driving force of angiogenesis [9]. Targeting EC metabolism instead of targeting growth factors and their receptors, such as VEGF and VEGFR, has achieved promising outcomes and has become a new therapeutic approach [10, 11].

Our previous study revealed that glucose metabolism may play an important role in the pathogenesis of IH [12]. 6-Phosphofructo-2-kinase/fructose-2,6-biphosphatase 3 (PFKFB3) is a metabolic enzyme that converts fructose-6-bisphosphate to fructose-2,6-bisphosphate (F-2,6-BP), which is the most potent allosteric activator of the rate-limiting enzyme phosphofructokinase-1 (PFK-1). Knockdown of PFK-1 can inhibit angiogenesis and reduce glycolytic flux in hemangioma-derived endothelial cells (HemECs) [12]. Recent studies have indicated that PFKFB3 is highly expressed in many cancers, such as breast and ovarian cancer, and plays a vital role in regulating different cellular events, including vessel sprouting, pathological angiogenesis and drug resistance [13, 14]. However, the role of PFKFB3 in IH is still largely unknown.

In the present study, we first used tissue microarray analysis to identify PFKFB3 as the most significantly differentially expressed gene (DEG). Then, treatment with PFK15, a selective inhibitor of PFKFB3 and genetic silencing with shPFKFB3 were used to study the effects of PFKFB3 inhibition on glucose metabolism and angiogenesis. We also investigated the role of PFKFB3 in a mouse model of tumorigenesis and found that targeting PFKFB3 can suppress IH angiogenesis and induce apoptosis.

## Methods

### Patients and samples

This study was approved by the Ethics Committee of the West China Hospital of Sichuan University. Informed consent was obtained for experimentation with human subjects from all the patients’ parents. All eight (three proliferating and five involuting) IH tissues were obtained surgically at our hospital (Additional file 1: Table S1), and the pathological diagnosis of IH was confirmed by hematoxylin–eosin (HE) and GLUT1 staining (Additional file 2: Fig. S1).

### Antibodies and reagents

Endothelial basal medium (EBM-2) containing supplements and growth factors was obtained from Lonza (Walkersville, MD, USA). EC medium (ECM) was obtained from ScienCell (San Diego, CA, USA). Fetal bovine serum (FBS), phosphate-buffered saline (PBS), penicillin and streptomycin were purchased from Gibco (Grand Island, NY, USA). Propranolol was purchased from Sigma (St. Louis, MO, USA). Antibodies against PFKFB3 and β-actin were obtained from Proteintech Biotechnology (Wuhan, China).

### Cell culture and treatment

HemEC isolation was performed as previously described [15]. HemECs were cultured in endothelial basal medium supplemented with 10% FBS plus penicillin (100 units/ml) and streptomycin (100 μg/ml). Cells at passages 3 to 8 were used for experiments. Cells were treated with 100 µM propranolol according to our previous research [15] and exposed to normoxia (21% O_2_) or hypoxia (1% O_2_).

### Tissue microarray analysis

As found in our previous study [12], tissue mRNA expression profiling was performed using the Affymetrix GeneChip Human Transcriptome Array 2.0 platform (Affymetrix, Inc., Santa Clara, CA, USA). Raw microarray data were extracted using Agilent Feature Extraction (v. 10.7), summarized, normalized and subjected to quality control using the GeneSpring GX program software (v. 12.6.1) package (Agilent Technologies) and R program. A microarray analysis was then performed using the Gene-Cloud of Biotechnology Information (GCBI, Shanghai, China; https://www.gcbi.com.cn) platform. Briefly, the significantly differentially expressed genes (DEGs) between the proliferating group and the involuting group were first identified based on an adjusted P value < 0.05 and fold change > 1.5 using SAM analysis. Hierarchical clustering was performed to display the expression profiles of DEGs between the two groups. GO enrichment and KEGG pathway analyses were then performed using the GCBI platform (https://www.gcbi.com.cn).

### Immunohistochemical analysis

Immunohistochemistry (IHC) was performed in accordance with our previous procedures [12]. Briefly, 5-μm tissue sections were cut, deparaffinized by heating to 60 °C for 1 h, subjected to three 15-min washes with xylene, rehydrated by consecutive washes in 100%, 95%, and 70% ethanol and washed once with water for 5 min. The sections were then incubated with 3% hydrogen peroxide for 30 min to inhibit endogenous peroxidase activity and subsequently underwent consecutive washes with PBS. Antigen retrieval was conducted by heating the sections twice for 20 min each in EDTA antigen retrieval buffer. The sections were blocked for 30 min in 5% serum at room temperature. Primary antibody against PFKFB3 (1:100, 13763-1-AP, Proteintech) was added for overnight incubation at 4 °C, and the slides were washed and incubated with secondary antibody at room temperature for 30 min. Images were acquired using a Leica microscope camera (Leica Microsystems, Wetzlar, Germany).

### Western blot analysis

Western blotting analysis was performed according to our previously described procedures [16]. Briefly, RIPA B lysis buffer with protease inhibitor cocktail was used for the harvesting of HemECs, and the protein concentration was determined using the Bradford protein assay kit. The protein samples were separated by sodium dodecyl sulfate–polyacrylamide gel electrophoresis (SDS‒PAGE) and electrophoretically transferred onto a nitrocellulose membrane. The membrane was blocked with TBST (10 mmol/L Tris–HCl, pH 7.4, 150 mmol/L NaCl, and 10% Tween 20) containing 5% (wt/vol) nonfat dry milk and incubated with primary antibody in TBST. The membrane was then washed three times and incubated with the appropriate secondary antibody. The protein bands were visualized by enhanced ECL-associated fluorography.

### CCK-8 cell proliferation assay

Briefly, 8000 HemECs were seeded into 96-well plates. The next day, the cells were treated with PFK15 for 24 h. After treatment, 10 μL per well of the CCK-8 kit reagent was added, and the plates were incubated for 2 h at 37 °C. Finally, the absorbance of each well at 450 nm was read using a microplate reader. All experiments were independently repeated at least three times.

### Transwell migration assay

HemECs (2 × 10^4^ cells/well) were plated in the top chamber (24-well insert; 8-μm pore size; Millipore, USA) in serum-free medium supplemented with EBM-2 medium plus 0.5% serum. The chamber was placed in a 24-well plate with 500 µL of EBM-2 and 10% serum supplemented with 100 µM propranolol and 5 µM PFK15 in the lower chamber. After 24 h, the bottom of the chamber insert was removed from the 24-well plate, wiped clean with a cotton swab, washed three times with PBS and then fixed with 4% paraformaldehyde for 30 min. The cell nuclei were stained with 0.1% crystal violet for quantification. Five random microscopic fields were selected to count the number of cells that migrated into the lower chamber. Each migration assay was conducted with at least 3 replicates.

### Flow cytometric analysis of cell apoptosis

A cell cycle detection kit purchased from 4A Biotech Co., Ltd. (Beijing, China), was used to detect the cell cycle. HemECs (4.0 × 10^5^/well) were plated in 6-well plates and cultured for 24 h. The cells were then collected by trypsinization, washed with cold PBS and immobilized with 95% cold ethanol overnight at 4 °C. The cells were washed again with PBS, incubated with RNase and then labelled with propidium iodide (PI) according to the manufacturer’s protocol. A CytoFLEX flow cytometer (Becton–Dickinson, USA) was used to detect the cell cycle. The cell cycle distribution was analyzed using ModFIT software (BD Biosciences).

### Glucose uptake assay

The uptake of glucose by HemECs was estimated using the Glucose Uptake Cell-Based Assay Kit (Cayman Chemical, USA). Briefly, HemECs (5 × 10^4^/well) were seeded in 96-well plates and then incubated overnight at 37 °C. The next day, the cells were treated for 1 h with glucose-free medium and fluorescent 2-NBDG at a concentration of glucose-free medium. The plate was centrifuged at room temperature for 5 min at 400 g, and the supernatant was then aspirated. Two hundred microliters of Cell-Based Assay Buffer was added to each well, and the cells were analyzed immediately using a CytoFLEX flow cytometer (Becton–Dickinson, USA).

### Lactate production

The lactate levels were measured using the L-Lactate Assay Kit (Cayman Chemical, USA) according to the manufacturer’s protocol. Briefly, 1 × 10^4^ cells/well in 120 μL of culture medium were seeded in a 96-well plate for 24 h. Then, 20 µL of medium was collected into a new 96-well plate for colorimetric detection at 490 nm with a microplate reader. All experiments were performed in triplicate.

### ATP production

The ATP levels were measured using an ATP Colorimetric/Fluorometric Assay kit (BioVision, USA) according to the manufacturer’s instructions. Briefly, lysates of 1 × 10^6^ cells were collected to detect the ATP concentrations using a microplate reader at 490 nm.

## Metabolic analysis

### Extraction of metabolites

After the addition of 300 μL of water, the samples were vortexed for 30 s. The samples were precooled in dry ice and subjected to three cycles of repeated freezing–thawing in liquid nitrogen. The samples were vortexed for 30 s and sonicated for 15 min in an ice-water bath. A 250 μL aliquot of the clear supernatant was transferred to a new EP tube, and 750 μL of methanol (precooled at − 40 °C) was then added. The remaining sample supernatant was used to measure the protein content. The samples were vortexed for 30 s, incubated at − 40 °C for 1 h and centrifuged at 12000 rpm (RCF = 13800(× g), R = 8.6 cm) for 15 min at 4 °C. A 900 μL aliquot of clear supernatant was collected and dried by spinning. The residue was reconstituted with 180 μL of ultrapure water, and the reconstituted samples were vortexed, filtered through the centrifuge tube filter, and subsequently transferred to inserts in injection vials for HPIC-MS/MS analysis.

### Standard solution preparation

Stock solutions were individually prepared by dissolving or diluting each standard substance to obtain a final concentration of 10 mmol/L. An aliquot of each of the stock solutions was transferred to a 10-mL flask to obtain a mixed working standard solution. A series of standard solutions for calibration were then prepared by stepwise dilution of the mixed standard solution.

### HPIC-MRM-MS analysis

HPIC separation was performed using an Thermo Scientific Dionex ICS-6000 HPIC System (Thermo Scientific) equipped with Dionex IonPac AS11-HC (2 × 250 mm) and AG11-HC (2 mm × 50 mm) columns. Mobile phase A was 100 mM NaOH in water, and mobile phase D was water. Another pumping system was used to supply the solvent (2 mM acetic acid in methanol), and the solvent was mixed with the effluent before entering the ESI (flow rate of 0.15 mL/min). The column temperature was set to 30 °C. The autosampler temperature was set to 4 °C, and the injection volume was 5 μL.

An AB SCIEX 6500 QTRAP + triple quadrupole mass spectrometer (AB Sciex) equipped with an electrospray ionization (ESI) interface was used for assay development. The typical ion source parameters were the following: ion spray voltage = − 4500 V, temperature = 450 °C, ion source gas 1 = 45 psi, ion source gas 2 = 45 psi, curtain gas = 30 psi.

The MRM parameters for each of the targeted analytes were optimized via flow injection analysis by injecting the standard solutions of the individual analytes into the API source of the mass spectrometer. Several of the most sensitive transitions were used in the MRM scan mode to optimize the collision energy for each Q1/Q3 pair. Among the optimized MRM transitions per analyte, the Q1/Q3 pairs that showed the highest sensitivity and selectivity were selected as the ‘quantifier’ for quantitative monitoring. The additional transitions acted as the ‘qualifier’ for the purpose of verifying the identity of the target analytes.

AB SCIEX Analyst Workstation software (1.6.3 AB SCIEX), MultiQuant 3.0.3 software and Chromeleon 7 were employed for MRM data acquisition and processing.

### RNA extraction

Total RNA was isolated and purified using TRIzol reagent (Invitrogen, Carlsbad, CA, USA) following the manufacturer’s procedure. The RNA amount and purity of each sample were quantified using a NanoDrop ND-1000 instrument (NanoDrop, Wilmington, DE, USA). The RNA integrity was assessed with a Bioanalyzer 2100 instrument (Agilent, CA, USA) based on RIN > 7.0 and confirmed by electrophoresis with a denaturing agarose gel. Poly(A) RNA was purified from 1 μg of total RNA using Dynabeads Oligo (dT)25-61005 (Thermo Fisher, CA, USA) via two rounds of purification. The poly(A) RNA was then fragmented into small pieces using a Magnesium RNA Fragmentation Module (NEB, cat., e6150, USA) at 94 °C for 5–7 min. The cleaved RNA fragments were then reverse-transcribed to create cDNA using SuperScript™ II Reverse Transcriptase (Invitrogen, cat., 1896649, USA), and the resulting cDNA was used to synthesize U-labelled second-stranded DNAs with *E. coli* DNA polymerase I (NEB, cat., m0209, USA), RNase H (NEB, cat., m0297, USA) and dUTP solution (Thermo Fisher, cat., R0133, USA). An A base was then added to the blunt ends of each strand to prepare them for ligation to the indexed adapters. Each adapter contained a T-base overhang for ligation of the adapter to the A-tailed fragmented DNA. Single- or dual-index adapters were ligated to the fragments, and size selection was performed with AMPure XP beads. After heat-labile UDG enzyme (NEB, cat. M0280, USA) treatment of the U-labelled second-stranded DNAs, the ligated products were amplified by PCR with the following conditions: initial denaturation at 95 °C for 3 min; 8 cycles of denaturation at 98 °C for 15 s, annealing at 60 °C for 15 s, and extension at 72 °C for 30 s; and final extension at 72 °C for 5 min. The average insert size for the final cDNA library was 300 ± 50 bp. We then performed 2 × 150-bp paired-end sequencing (PE150) using an Illumina NovaSeq^™^ 6000 (LC-Bio Technology Co., Ltd., Hangzhou, China) following the vendor’s recommended protocol.

### RNA-seq data analysis

Fastp software (https://github.com/OpenGene/fastp) was used to remove the reads that contained adaptor contamination, low-quality bases and undetermined bases with the default parameters. The sequence quality was then also verified using fastp. We used HISAT2 (https://ccb.jhu.edu/software/hisat2) to map reads to the *Homo sapiens* GRCh38 reference genome, and the mapped reads of each sample were assembled using StringTie (https://ccb.jhu.edu/software/stringtie) with default parameters. All transcriptomes from all the samples were then merged to reconstruct a comprehensive transcriptome using gffcompare (https://github.com/gpertea/gffcompare/). After the final transcriptome was generated, StringTie was used to determine the expression levels of mRNAs by calculating the FPKM values (FPKM = [total_exon_fragments/mapped_reads (millions) × exon_length (kB)]). The differentially expressed mRNAs were selected based on a fold change (FC) > 2 or < 0.5 and by performing a parametric F test comparing nested linear models (p value < 0.05) with an R package.

### Integrated analysis of the metabolomic and transcriptomic data

For further integrative analysis of the transcriptomics and metabolomics data at the pathway level, all the DEGs and differentially expressed metabolites (DEMs) were used to perform pathway analysis with MetaboAnalyst 5.0 (https://www.metaboanalyst.ca/) [17, 18].

### ROS detection

Briefly, HemECs were seeded in 6-well plates, and the next day, the cells were treated with propranolol and PFK15 for 24 h. The cells were then incubated with CDFH-DA, washed three times with PBS, observed using a fluorescence microscope (Olympus) and measured with a flow cytometer (Beckman).

### Transmission electron microscopy (TEM) analysis

HemECs were seeded in 6-cm culture dishes, and the next day, the cells were treated with propranolol and PFK15 for 24 h. A morphological examination of the propranolol-treated HemECs was then performed with a transmission electron microscope (CM12 Philips, Amsterdam, Netherlands). The samples were stained with 2% (w/v) phosphotungstic acid and placed on copper grids for TEM observation.

### Immunoprecipitation

The cells were lysed with protein lysis extract buffer and then centrifuged at 12000 × g for 10 min at 4 °C, and the supernatant was then collected. Eighty microliters of supernatant was then removed and used as input. The supernatant was incubated with the indicated antibodies and protein-A-agarose overnight at 4 °C. Isotype-matched IgG was used as a negative control. The beads were washed three times with ice-cold buffer and decoupled by boiling in sodium dodecyl sulfate (SDS) loading buffer. The samples were analyzed by western blotting.

### Lentiviral vector transfection

Briefly, specific shRNA for PFKFB3 was purchased from GeneChem Co., Ltd. (Shanghai, China), and transfection was performed using Lipofectamine 2000 (Invitrogen, USA) according to the manufacturer’s protocol. Forty-eight hours after transfection, the cells were collected for various experiments.

### Mouse model of IH

The in vivo study was approved by the Ethics Committee of West China Hospital of Sichuan University. To investigate the role of PFKFB3 inhibition by PFK15, the murine hemangioma model was established by subcutaneous injection of HemECs and hemangioma-derived pericytes (HemPCs). HemPCs (2 × 10^6^) and HemECs (2 × 10^6^) were mixed in 200 μL of Matrigel (BD, San Jose, CA, USA) and subcutaneously administered into the backs of BALB/C-nu mice (aged 4 weeks, male, purchased from Chinese Academy of Science, Shanghai) for 7 days. Four groups (five mice per group) were then treated with PBS (control), propranolol alone (10 mg/kg), PFK15 alone (25 mg/kg), or propranolol in combination with PFK15 every other day for 7 days by intratumoral injection. To investigate the role of PFKFB3 inhibition by shRNA, the mice were injected subcutaneously with 2 × 10^6^ PFKFB3-knockout HemECs and HemPCs (2 × 10^6^) for 14 days.

After the treatment, the experimental animals were sacrificed, and the tumors of the experimental animals were dissected.

### Microvessel destiny (MVD) assay

To analyze the effect of different formations of PFK15 on the growth of microvessels, an MVD assay was performed. Paraffin-embedded sections of Matrigel explants were stained with hematoxylin and eosin (HE). For assessment of the MVD, 4 fields from mid-Matrigel HE-stained sections of each of the four animals in the group were analyzed. The microvessels were quantified by counting the luminal structures containing red blood cells [19]. Other paraffin-embedded sections of Matrigel explants were utilized for immunohistochemical staining for CD31.

### Statistical analysis

The data were analyzed using SPSS version 23.0 (SPSS, Chicago, IL, USA). Continuous variables are presented as the means ± standard deviations, and Student’s t test was used to assess the differences between two groups. P values < 0.05 were considered statistically significant.

## Results

### PFKFB3 inhibition by PFK15 suppresses HemEC angiogenesis

**Fig. 1:**
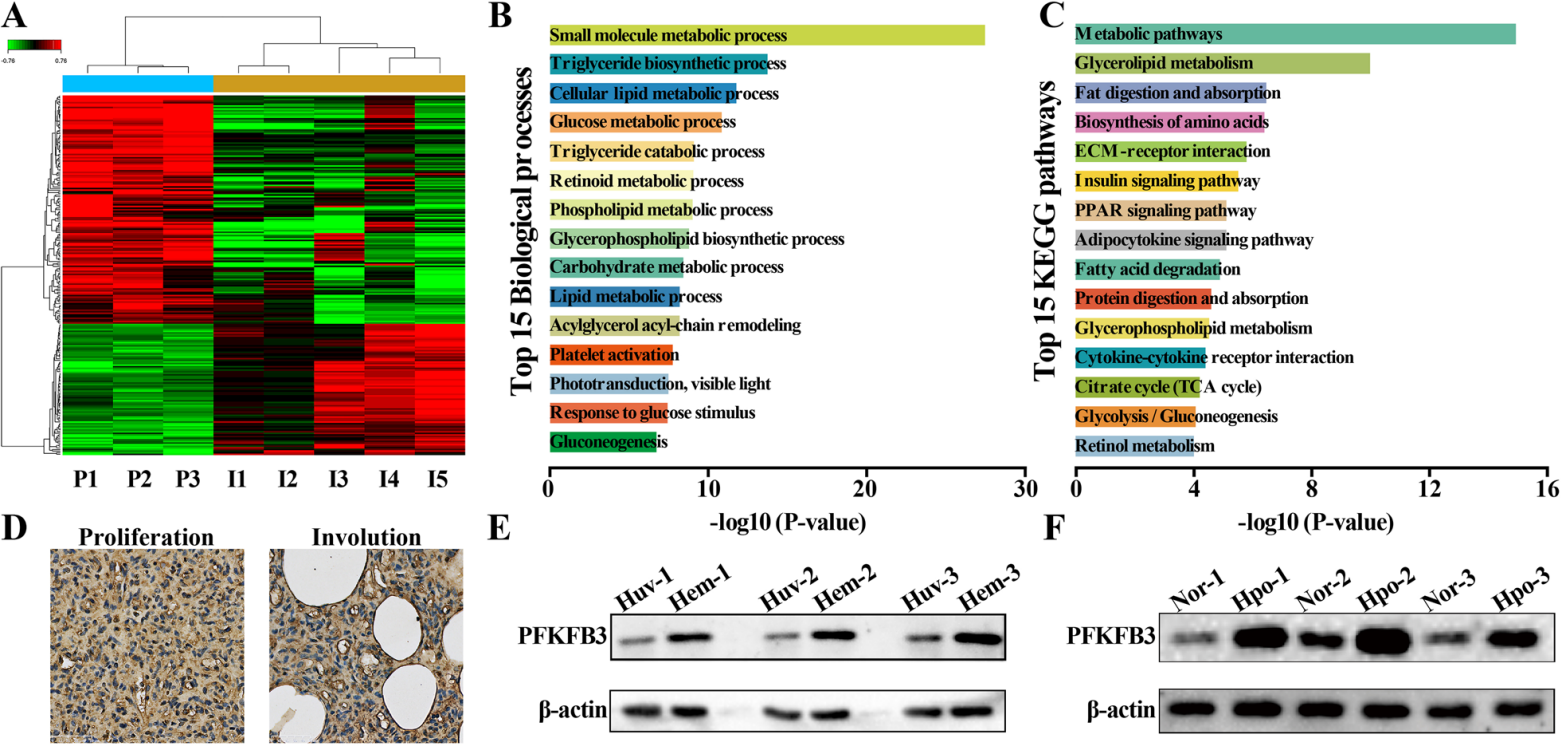
PFKFB3 overexpression in proliferating IH tissues and HemECs. **A**, Heatmap of the 222 differentially expressed genes (DEGs). **B**, Top 15 Gene Ontology terms enriched in the DEGs. **C**, Top 15 pathway terms enriched in the DEGs. **D**, PFKFB3 protein expression in proliferating and involuting IH tissues was detected by immunohistochemistry. **E**, PFKFB3 protein expression in hemangioma-derived endothelial cells (HemECs) and human umbilical vein endothelial cells (HUVECs) was measured by western blotting. **F**, PFKFB3 protein expression in hypoxia and normoxia was measured by western blotting. Nor, normoxia; Hpo, hypoxia

A total of 222 DEGs—141 upregulated and 81 downregulated—were identified, as shown in the heatmap (Fig. 1A). PFKFB3 was one of the most significant DEGs (Additional file 4: Table S2). Then, GO term and KEGG pathway analyses were performed to explore the roles of all DEGs. The DEGs were enriched mostly in cell metabolism-related biological processes, including ‘cellular lipid metabolic process’, ‘glucose metabolic process’, and ‘gluconeogenesis’ (Fig. 1B). Similarly, metabolism-related pathways were the predominantly enriched KEGG pathways, for example, ‘glycerolipid metabolism’, ‘fatty acid degradation’, ‘citrate cycle’ and ‘glycolysis/gluconeogenesis’ (Fig. 1C). Subsequently, IHC was used to validate the microarray results, and the expression of PFKFB3 was found to be higher in proliferating IH tissues than in involuting IH tissues (Fig. 1D). In addition, PFKFB3 protein expression was higher in HemECs than in human umbilical vein endothelial cells (HUVECs) (Fig. 1E). It is widely accepted that hypoxia is considered an independent important pathogenic factor for the development of IH [20]. In addition, the hypoxia-inducible factor1α (HIF1α) pathway is one of the most important signaling pathway regulating IH development [4]. Moreover, hypoxia can increase the expression of glycolytic enzymes to enhance the glycolytic flux in EC [21]. In the present study, hypoxia increased the protein expression of PFKFB3 in HemECs compared with the level found in HUVECs (Fig. 1F). Taken together, the above results indicated that cell metabolism, particularly glucose metabolism, is closely related to the development of IH and that PFKFB3 is overexpressed in proliferating IH tissues and HemECs and may play a vital role in the regulation of IH development.

### PFK15 inhibits HemEC angiogenesis

**Fig. 2:**
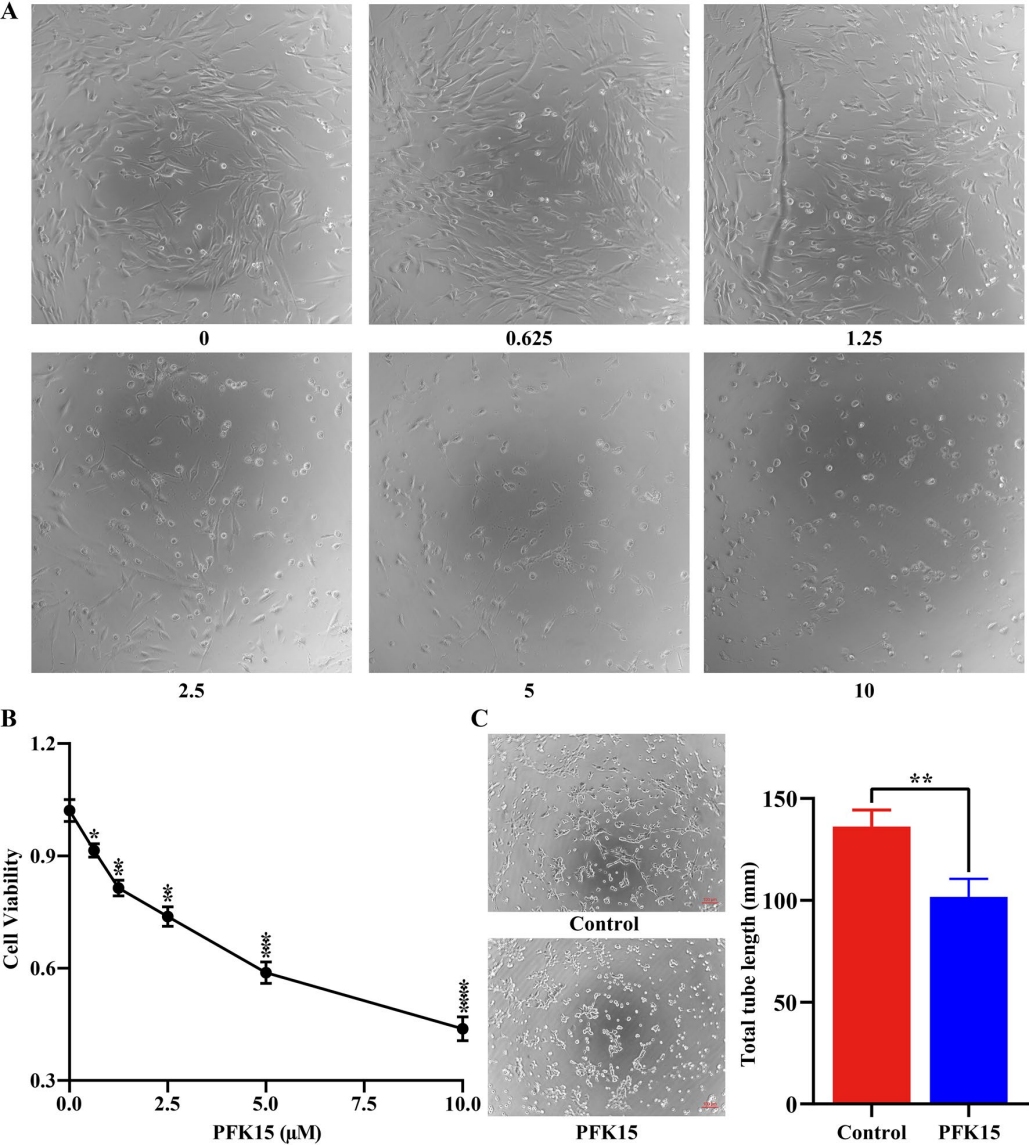
PFK15 inhibits HemEC angiogenesis. **A**, Morphological alterations after PFK15 treatment in HemECs. **B**, Effects of PFK15 on HemEC proliferation. **C**, Effects of PFK15 on HemEC tube formation in vitro

A CCK-8 assay was used to investigate the ability of PFK15, a selective inhibitor of PFKFB3, to inhibit HemEC proliferation in vitro. With increasing drug concentration, PFK15 treatment induced progressive morphological alterations in HemECs, including a decrease in the cell density, cell shrinkage and an increase in cell fragmentation (Fig. 2A), indicating that PFK15 decreased the viability of HemECs in a dose-dependent manner (Fig. 2B). After calculation of the IC50 following 24 h of continuous PFK15 exposure (IC50 = 6.75 µM), 5 µM PFKF15 was selected as the treatment concentration for further experiments. To further validate the role of PFK15 in the inhibition of cell proliferation, HemEC tube formation was evaluated in vitro. The results demonstrated that PFK15 significantly suppressed angiogenesis, as determined by the decrease in tube formation compared to that in the control group (Fig. 2C). In summary, our results indicated that inhibition of PFKFB3 with PFK15 can inhibit HemEC angiogenesis.

### PFK15 suppresses HemEC glucose metabolism

**Fig. 3:**
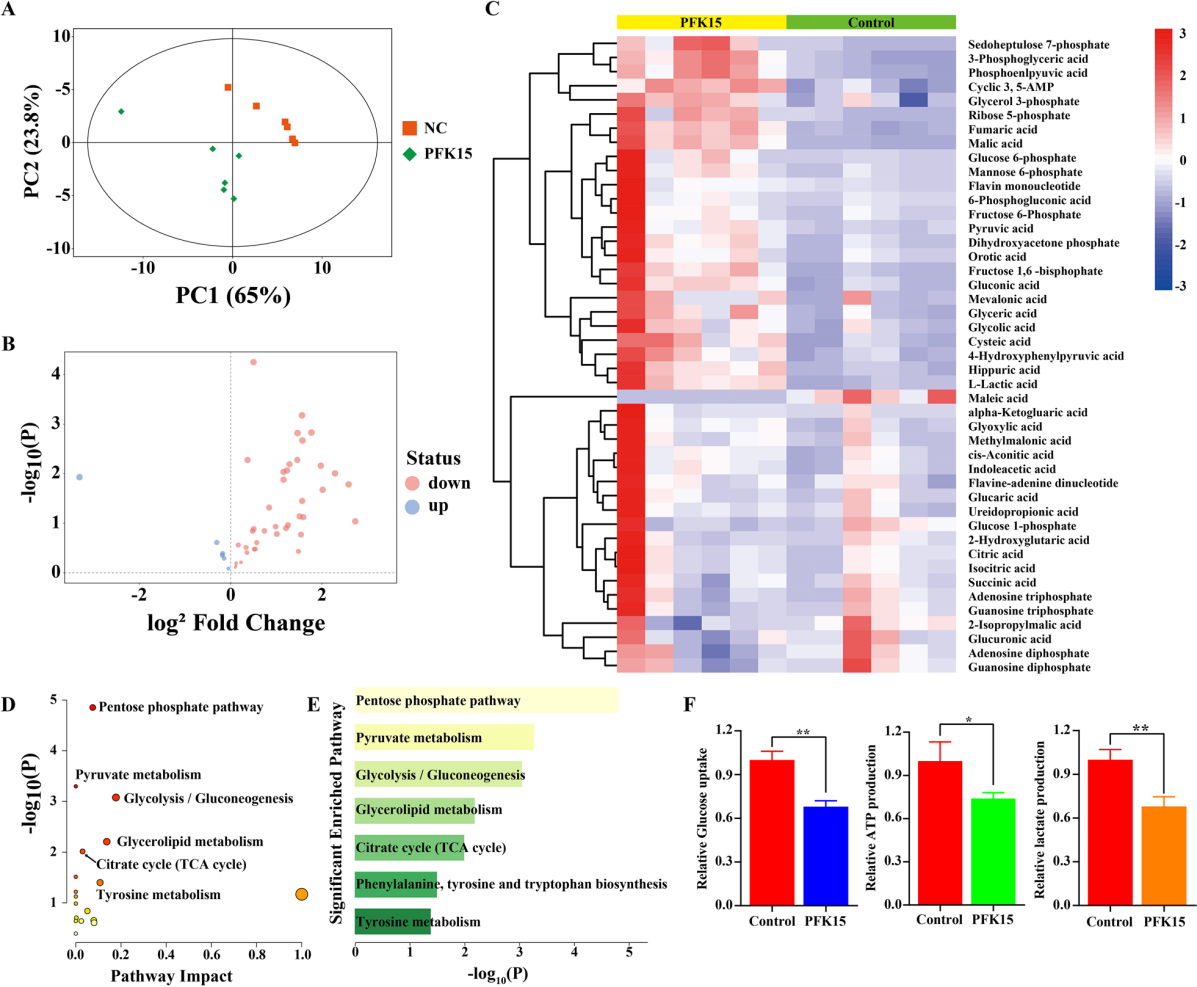
PFK15 suppresses HemEC glucose metabolism. **A**, Score plot from the principal component analysis between control and PFK15-treated cells, as revealed by a metabolic analysis. B, Volcano plot of significantly altered metabolites. **C**, Heatmap of identified metabolites. **D**, **E**, Pathways significantly enriched in differentially expressed metabolites. F, Inhibition of glycolytic flux in HemECs after PFK15 treatment

First, targeted metabolomic analysis of glucose was carried out to investigate the effect of PFK15 on HemEC glucose metabolism. The principal component analysis (PCA) (Fig. 3A) score plots showed significant separation between the control and PFK15-treated groups, reflecting considerable metabolic differences between these two groups. As shown in the volcano plot (Fig. 3B), a total of 45 metabolites were identified and are visualized on the hierarchical clustering analysis heatmap in Fig. 3C. Interestingly, many glycolytic metabolites, including glucose 6-phosphate, fructose 6-phosphate, and fructose 1,6-bisphosphate, were still more abundant after inhibition of PFKFB3 than in the control group (Fig. 3C). In addition, metabolites belonging to ‘citrate cycle (TCA cycle)’, including citric acid, isocitric acid, fumaric acid, alpha-Ketoglutaric acid, malic acid and succinic acid, were also significantly increased after PFKFB3 inhibition compared with their levels in the control group (Fig. 3C). The most significant metabolites were then used for KEGG pathway analysis to understand their roles based on a threshold of Q < 0.05. In total, 14 metabolites were selected (Additional file 5: Table S3), and these metabolites were enriched mainly in glycolysis-related pathways, such as ‘pentose phosphate pathway’, ‘glycolysis/gluconeogenesis’, ‘pyruvate metabolism’ and ‘TCA cycle’(Fig. 3D, E). These results showed that PFK15 could affect glycolysis in HemECs. The glycolytic activity of HemECs was then measured by measuring glucose uptake, the intracellular ATP level and lactate production after PFK15 treatment. As shown in Fig. 3F, PFK15 treatment decreased the uptake of glucose, the production of lactate and the intracellular ATP level. Taken together, our data showed that inhibition of PFKFB3 with PFK15 suppressed glucose metabolism of HemECs.

### PFK15 inhibited HemEC migration and induced apoptosis

**Fig. 4:**
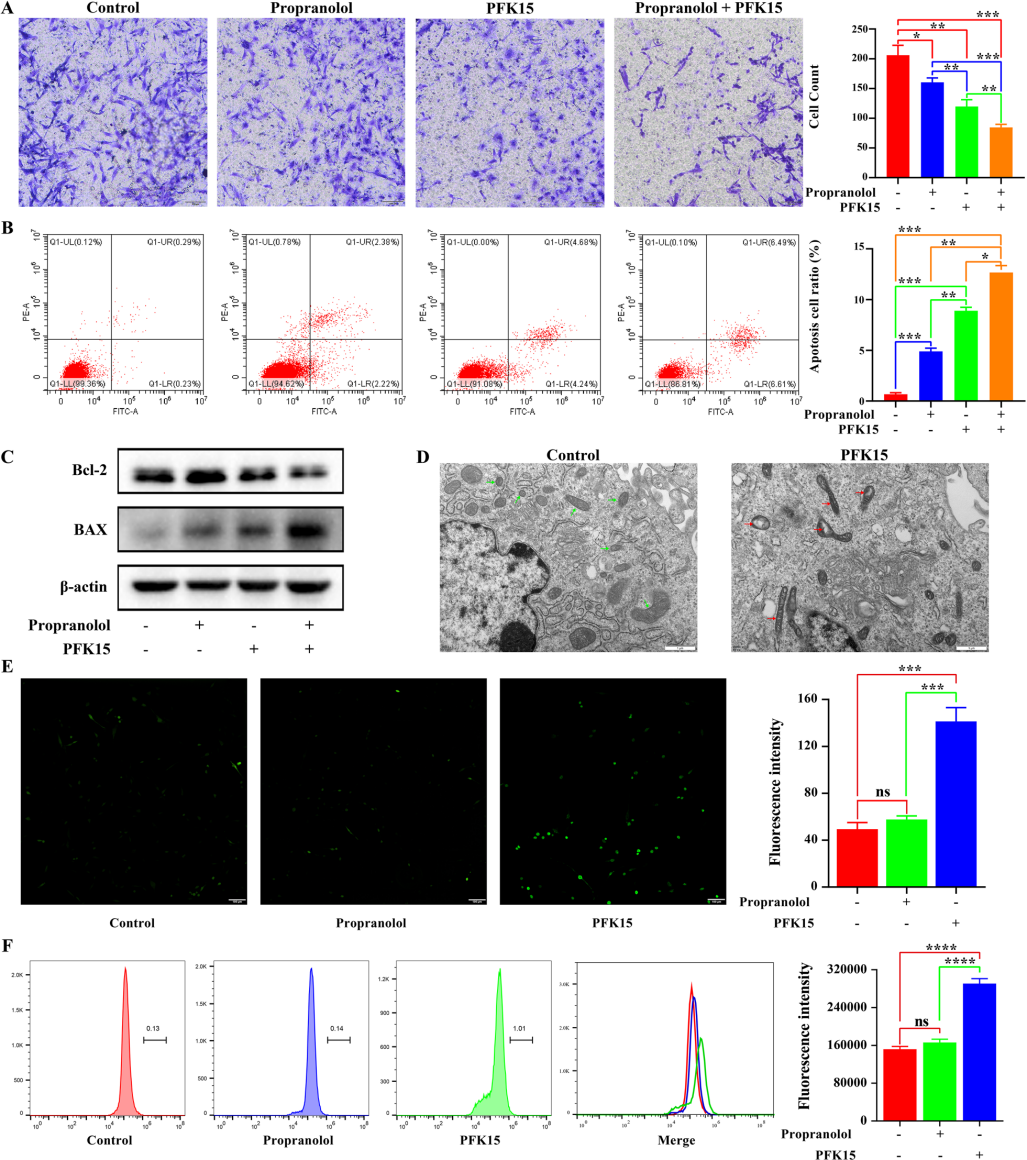
PFK15 inhibits HemEC migration and induces apoptosis. **A**, Effects of PFK15 on HemEC migration. *, P < 0.05; **, P < 0.01; ***, P < 0.001. **B**, Effects of PFK15 on HemEC apoptosis. *, P < 0.05; **, P < 0.01; ***, P < 0.001. **C**, Apoptosis pathway-related protein expression after PFK15 treatment. **D**, Effects of PFK15 on ultrastructural changes in HemEC mitochondria. **E**, Effects of PFK15 on reactive oxygen species (ROS) production, as evaluated by fluorescence microscopy. ***, P < 0.001; ns, no significance. **F**, Effects of PFK15 on ROS production, as evaluated by flow cytometry. ****, P < 0.0001; ns, no significance

A high rate of glycolysis is required to support tumor-activated EC proliferation and migration to maintain angiogenesis [9]. Therefore, we examined the potential role of PFK15 in cell migration. The Transwell assay results revealed that PFK15 suppressed the migration of HemECs (Fig. 4A). In addition, a synergistic inhibitory effect was observed when PFK15 treatment was combined with propranolol treatment (Fig. 4A). To determine whether the synergistic migration inhibition induced by PFK15 was due to apoptosis, flow cytometric analysis using Annexin V and PI labelling was performed. The results showed a larger apoptotic cell population in the PFK15-treated group than in the control group (Fig. 4B). Similarly, cotreatment with PFK15 and propranolol led to a significant increase in the percentage of apoptotic HemECs (Fig. 4B). Furthermore, upregulation of Bax and downregulation of Bcl-2 protein expression was observed by western blot analysis after PFK15 treatment and the combination treatment in HemECs (Fig. 4C). In most cells, apoptosis is coordinated in mitochondria by the Bcl-2 protein family [22]. Therefore, ultrastructural changes in HemEC mitochondria after PFK15 treatment were investigated by TEM. Significant mitochondrial stress responses were observed, including an increased mitochondrial matrix density, collapsed mitochondrial cristae, mitochondrial swelling and mitochondrial vacuolar degeneration, after PFK15 treatment compared to control treatment (Fig. 4D). Mitochondrial stress responses can greatly increase the production of reactive oxygen species (ROS), resulting in activation of the mitochondrial apoptotic pathway [23]. The results showed increased ROS production after PFK15 treatment compared to that in the control and propranolol groups (Fig. 4E, F). In summary, targeting PFKFB3 with PFK15 inhibited cell migration and activated the mitochondrial apoptosis pathway.

### PFKFB3 knockdown suppresses HemEC angiogenesis

**Fig. 5:**
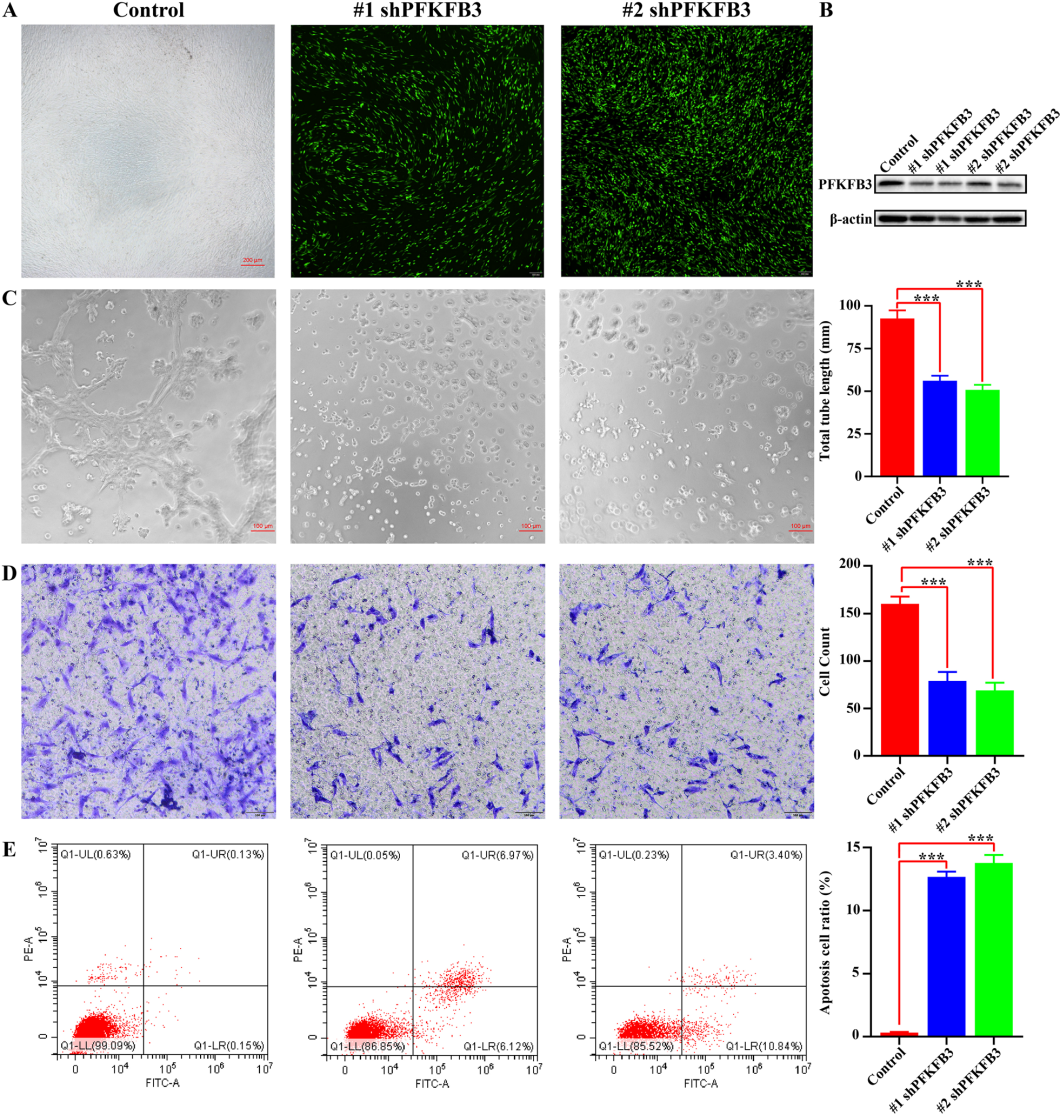
PFKFB3 knockdown suppresses HemEC angiogenesis and induces apoptosis **A**, Lentiviral vectors expressing shRNA targeting PFKFB3. **B**, Protein expression level of PFKFB3 after transduction of shPFKFB3. **C**, Effect of shPFKFB3 on tube formation in vitro. ***, P < 0.001. **D**, Effect of shPFKFB3 on cell migration in vitro. ***, P < 0.001. **E**, Effect of shPFKFB3 on apoptosis. ***, P < 0.001

To further investigate whether the PFKFB3 level affects angiogenic activity, we constructed lentiviral vectors expressing shRNA targeting PFKFB3 (Fig. 5A). The protein expression level of PFKFB3 in HemECs decreased significantly after transfection with shPFKFB3 (Fig. 5B). Knockdown of PFKFB3 also significantly impaired the tube formation of HemECs (Fig. 5C). Cell migration was significantly inhibited by shPFKFB3 (Fig. 5D). In addition, the proportion of apoptotic cells was higher in the shPFKFB3 lentivirus group than in the control group (Fig. 5E). In summary, the abovementioned results demonstrated that PFKFB3 knockdown significantly suppressed HemEC angiogenesis.

### PFKFB3 inhibition downregulated activation of the PI3K/Akt signaling pathway in HemECs

**Fig. 6:**
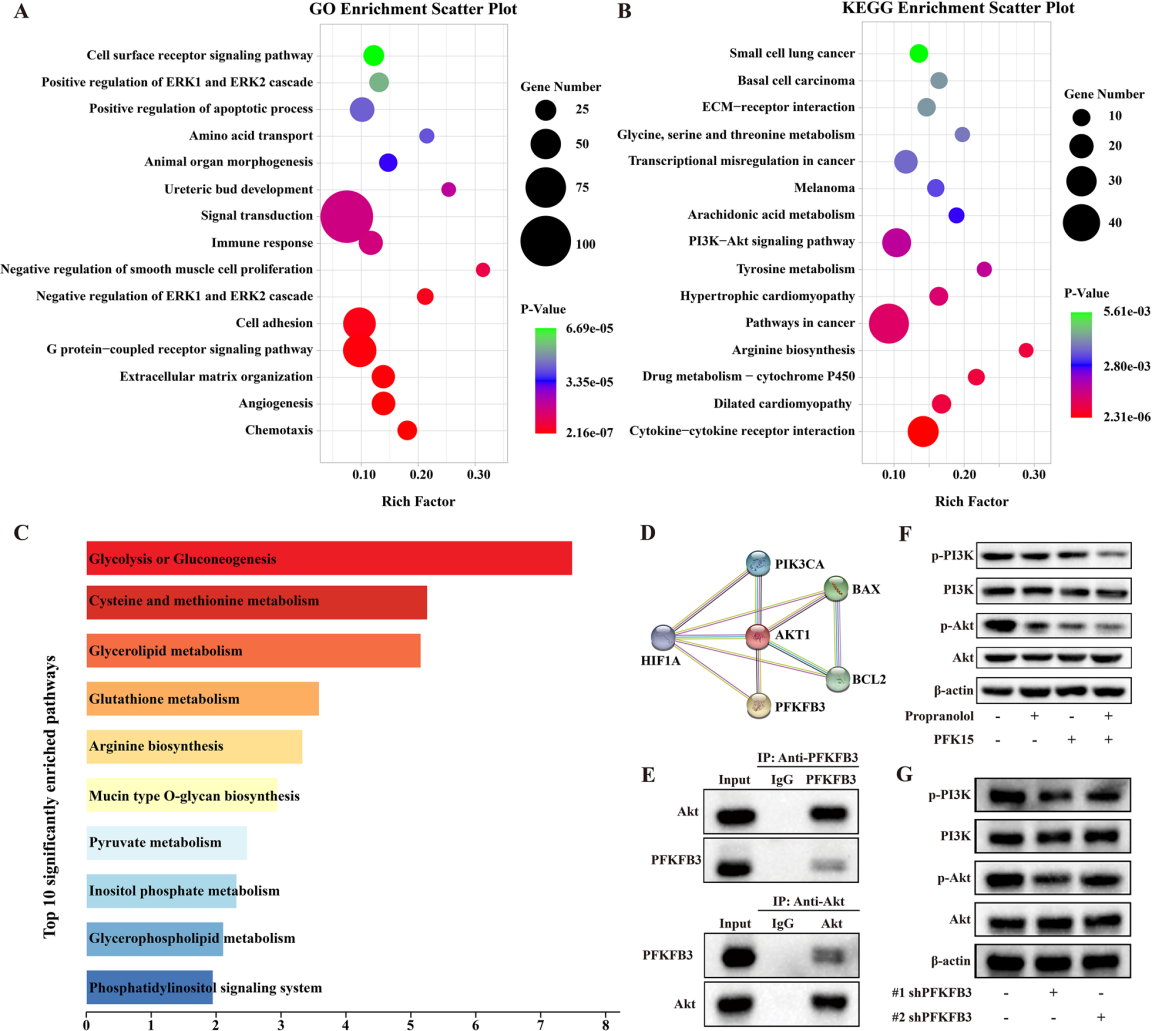
PFKFB3 inhibition inhibits the activation of the PI3K/Akt signaling pathway in HemECs. **A**, Top 15 Gene Ontology terms enriched in the differentially expressed genes (DEGs) between the PFK15 treatment and control groups. **B**, Top 15 KEGG pathways enriched in the DEGs between the PFK15 treatment and control groups. **C**, Top 10 significantly enriched pathways identified by integrated transcriptional and metabolic analysis. **D**, STRING functional protein associations based on transcriptional data. **E**, Immunoblot analysis of Akt and PFKFB3. **F**, PI3K-Akt pathway protein expression after PFKFB3 inhibition by PFK15. **G**, PI3K-Akt pathway protein expression after PFKFB3 inhibition by shPFKFB3

To identify the key pathway regulating angiogenic activity in IH after PFKFB3 inhibition, transcriptional analysis was performed between the PFK15-treated group and the control group. In total, 1240 DEGs (357 upregulated and 783 downregulated) were identified, as shown in the bar plot (Additional file 3: Fig. S2a) and volcano plot (Additional file 3: Fig. S2b). In addition, a heatmap visualizing the hierarchical clustering analysis results was generated for the top 100 DEGs (Additional file 3: Fig. S2c). A GO analysis then revealed that these DEGs were enriched mostly in the biological process terms ‘angiogenesis’, ‘cell adhesion’ and ‘positive regulation of apoptotic process’ (Fig. 6A). KEGG pathway analysis showed that the PI3K-Akt signaling pathway was significantly enriched, in addition to cell metabolism-related pathways, including ‘arginine biosynthesis’, ‘tyrosine metabolism’, and ‘glycine, serine and threonine metabolism’ (Fig. 6B). These data indicated that the PI3K-Akt pathway may participate in the regulation of angiogenesis and cell metabolism in IH after PFK15 treatment. In addition, integrated analysis of the transcriptional and metabolic results was performed to further determine the changes in and associations of metabolites and genes. The results showed that ‘glycolysis or gluconeogenesis’, ‘cysteine and methionine metabolism’, ‘glycerolipid metabolism’ and ‘glutathione metabolism’ were the most significant pathways (Fig. 6C), suggesting that inhibition of PFKFB3 mainly influences glycolysis in HemECs.

To further show the role of the PI3K-Akt pathway in the regulation of PFKFB3, functional protein association analysis was first performed with STRING to establish a potential relationship between Akt and PFKFB3. PFKFB was directly linked to Akt, which also had a close association with Bax and Bcl-2 (Fig. 6D). Next, Co-IP was used to validate the protein‒protein interaction between PFKFB3 and Akt. The Akt protein could be precipitated from HemEC extracts with the anti-PFKFB3 antibody (Fig. 6E); reciprocally, the anti-Akt antibody specifically immunoprecipitated the PFKFB3 protein (Fig. 6F).

Because the PI3K/Akt signaling pathway has been implicated in the regulation of PFKFB3, we sought to determine whether PFK15 induces HemEC apoptosis through this pathway. The western blot results showed that PFK15 decreased the phosphorylation of both PI3K and Akt (Fig. 6F). Compared with treatment with each agent alone, cotreatment with PFK15 and propranolol led to further reductions in the phosphorylation of PI3K/Akt (Fig. 6G). Additionally, silencing PFKFB3 with shPFKFB3 significantly reduced the phosphorylation of PI3K and Akt. Hence, our results demonstrated that inhibition of PFKFB3 suppressed the PI3K-Akt signaling pathways and induced apoptosis.

### Suppression of PFKFB3 inhibits IH vessel formation in vivo

**Fig. 7:**
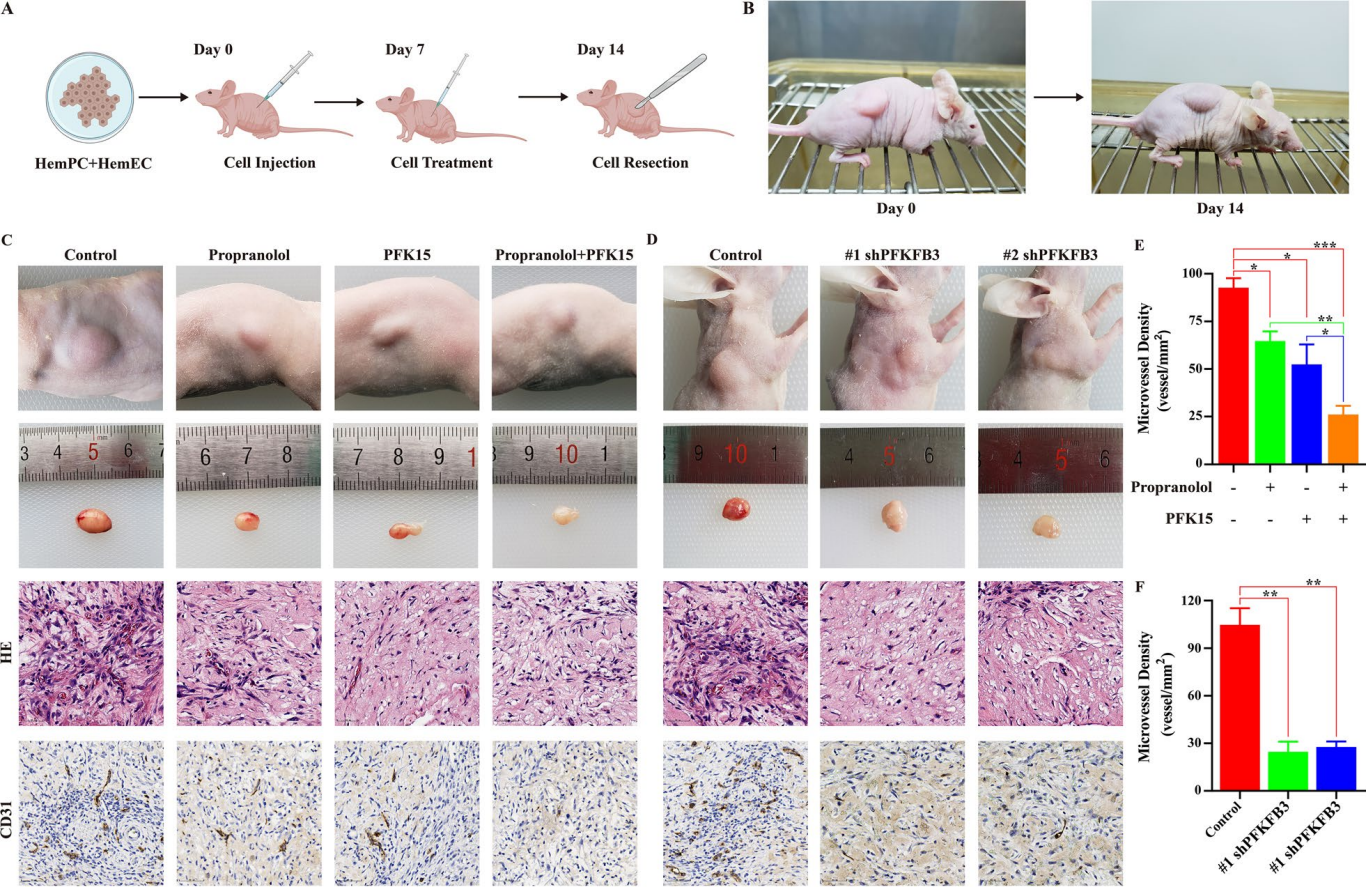
Suppression of PFKFB3 inhibits IH vessel formation in vivo. **A**, Diagram of the mouse xenograft models. **B**, Photographs of tumors in the animal model 14 days after cell implantation. **C**, Photographs of HE and CD31 staining of hemangiomas and xenografts in animals belonging to the control group, propranolol group, PFK15 group, and combination group. **D**, Photographs of HE and CD31 staining of hemangiomas and xenografts in animals belonging to the control and shPFKFB3 groups. **E**, MVD values of the control group, propranolol group, PFK15 group, and combination group. *, P < 0.05; **, P < 0.01; ***, P < 0.001. **F**, MVD values of the control group and shPFKFB3 group. **, P < 0.01

To investigate whether our in vitro findings could be translated into an in vivo setting, mouse xenograft models were established using HemECs and hemangioma-derived pericytes (Fig. 7A). At day 14, the experimental animals were sacrificed, and the tumors of the experimental animals were dissected (Fig. 7B). Significant reductions in tumor growth were observed on day 14 in both the PFK15-only and combination groups (Fig. 7C). The HE staining results demonstrated that PFK15 alone or in combination with propranolol significantly reduced the number of microvessels (Fig. 7C). In addition, PFK15 alone or in combination with propranolol decreased CD31 expression compared to that in the control group (Fig. 7C). Moreover, transduction with shPFKFB3 reduced the number of microvessels and the expression of CD31 (Fig. 7D). The microvessel density (MVD) was lower in the PFK15-treated group than in the control group and was the lowest in the combination treatment group (Fig. 7E). Silencing PFKFB3 also significantly decreased the MVD (Fig. 7F). All these results demonstrated that suppression of PFKFB3 inhibited IH vessel formation in vivo.

## Discussion

Although the exact pathogenesis of IH is not clear, angiogenesis plays a vital role in IH development [24]. Angiogenesis is defined as the formation of new blood vessels sprouting from the preexisting vasculature. ECs must meet the bioenergetic and biomass demands of cell proliferation and migration during sprouting angiogenesis.

Glucose, the main metabolic substrate for EC energy production, is metabolized mainly by glycolysis rather than oxidative phosphorylation (OXPHOS) in ECs [11]. Compared to OXPHOS, glycolysis in ECs has several advantages, including a higher rate of ATP production, reduced production of ROS, and increased biomass synthesis to maintain EC proliferation and migration [9]. In addition, targeting the key glycolytic enzyme PFKFB3 decreases the growth and migration of ECs in vitro and normalizes abnormal tumor vessels in vivo [25, 26]. Recent studies showed that key glycolytic enzymes, including hexokinase 2 (HK2), PFKFB3, PKM2 and LDHA, are more highly expressed in proliferating IH tissues than in involuting IH tissues [27]. Our previous study showed that glucose metabolism-related pathways, such as ‘OXPHOS’, ‘pyruvate metabolism’ and ‘TCA cycle’, were associated with the development of IH [12]. In addition, the expression of PFK-1, the most important rate-limiting enzyme in glycolysis, was significantly higher in proliferating IH tissue and HemECs [12]. Moreover, silencing PFK-1 suppressed HemEC proliferation and migration, reduced glucose uptake and decreased lactate and ATP production [12]. These results indicate that glucose metabolism could be tightly linked to the pathogenesis of IH. Therefore, we hypothesized that glycolysis may participate in the development of IH [28]. However, the mechanism by which glycolysis regulates HemEC sprouting angiogenesis is still largely unknown.

The PI3K-Akt pathway is one of the most commonly activated pathways in human tumors [29]. Under stimulation by insulin, growth factors and cytokines, the PI3K-Akt pathway is activated to regulate key cell metabolic processes, including glucose metabolism, biosynthesis of macromolecules and maintenance of redox homeostasis, to meet the metabolic demands for supporting the growth of individual cells [30]. This pathway can directly control glucose metabolism through phosphorylation-mediated regulation of metabolic enzymes to promote glycolysis [31]. Akt phosphorylation can promote the translocation of GLUT1 to the plasma membrane and then increase glucose uptake [32, 33]. In addition, Akt can phosphorylate and activate specific key glycolytic enzymes, including HK2 [34], PFKFB3 [35] and PFK1 [36], to promote high glycolytic flux. Moreover, the PI3K-Akt pathway enhances glycolysis through activation of the downstream transcription factor hypoxia-inducible factor 1α (HIF1α), which induces the upregulation of GLUT1 and nearly all key glycolytic enzymes [37]. In the present study, inhibition of PFKFB3 not only suppressed HemEC proliferation and migration but also decreased glycolytic flux and influenced glucose metabolism in HemECs. In addition, downregulation of PI3K and Akt expression was found after PFKFB3 inhibition. Moreover, a direct association between Akt and PFKFB3 in HemECs was validated by Co-IP. These results revealed a protein–protein interaction between PFKFB3 and Akt and potential regulation of the PI3K-Akt pathway by PFKFB3. Our previous study demonstrated that the PI3K-Akt signaling pathway participates in the regulation of angiogenesis in IH [4, 16]. Taken together, these data demonstrated a protein–protein interaction between PFKFB3 and Akt, indicating that PFKFB3 inhibition could regulate the PI3K/Akt pathway to affect glucose metabolism and angiogenesis in HemEC.

Apoptosis, or programmed cell death, is a process by which infected, damaged or unwanted cells are removed to maintain cell homeostasis. There are two major apoptotic pathways: the extrinsic (death receptor) pathway and the intrinsic (mitochondrial) pathway [38]. As the master regulators of apoptosis, Bcl-2 family proteins comprise antiapoptotic proteins (including Bcl-2 and Bcl-XL) and proapoptotic proteins (such as Bax and Bak). Under various proapoptotic stimuli, the antiapoptotic protein Bcl-2 is inhibited, and the proapoptotic protein Bax is activated, resulting in mitochondrial outer membrane permeabilization (MOMP). Once MOMP occurs, cytochrome *c* is released into the cytosol and then further activates caspases to finally induce cell death [38]. In the present study, upregulation of the Bax protein associated with downregulation of the Bcl-2 protein was observed in HemECs after PFKFB3 inhibition. In addition, mitochondrial stress responses, including an increased mitochondrial matrix density, collapsed mitochondrial cristae, mitochondrial swelling and mitochondrial vacuolar degeneration, were observed. These results indicated that intrinsic mitochondrial apoptosis occurred after PFKFB3 inhibition.

However, the underlying mechanism by which PFKFB3 inhibits apoptosis is unknown. It is widely accepted that excess levels of ROS can induce mitochondrial stress and promote apoptosis [39]. Endogenous ROS production results mainly from leaks during mitochondrial electron transport chain activity during OXPHOS. In our study, the metabolic analysis results showed that ‘TCA cycle’ was one of the most significantly enriched pathways. Additionally, the ROS level was higher in the PFK15-treated group than in the control group or propranolol-treated group. These results showed that ROS production was increased after PFKFB3 inhibition. Moreover, ‘glutathione metabolism’ was significantly enriched, as shown by integrated analysis of transcriptional and metabolic data. Glutathione (GSH), a major antioxidant within cells, can directly scavenge a wide variety of reactive species to reduce damage from ROS [40]. At the expense of NADPH generated via glucose metabolism through the pentose phosphate pathway, GSH reductase can reduce glutathione disulfide (GSSG) to GSH. When oxidative stress occurs, GSH is oxidized to GSSG by glutathione peroxidase 1, accompanied by loss of its biological activity, resulting in intracellular GSH depletion, an early hallmark in the progression of cell death [40]. Therefore, we speculated that inhibition of PFKFB3 may suppress the expression of glutathione peroxidase 1 to induce GSH depletion and result in apoptosis. To further investigate the mechanism of HemEC apoptosis under PFKFB3 inhibition, the role of glutathione peroxidase 1 in the regulation of ROS to control apoptosis will be investigated in our next study.

In recent decades, the main antiangiogenic strategy has been to inhibit proangiogenic factors in order to starve cancer cells, eliciting the arrest of tumor vessel growth. However, insufficient efficacy due to adaptive and compensatory mechanisms activated after VEGF/VEGFR pathway blockade limits the overall success of VEGF-targeted antiangiogenic drugs. A hallmark of tumors, glucose metabolic reprogramming (aerobic glycolysis) is more highly activated than in quiescent ECs under both physiological and pathological conditions. Several clinical trials of key glycolytic enzyme inhibitors in the treatment of different tumors are currently enrolling subjects [41]. Regarding IH treatment, targeting FPFKB3 not only suppresses glycolysis but also induces HemEC apoptosis to inhibit angiogenesis. Therefore, we speculate that targeting HemEC glucose metabolism may be a promising treatment strategy for IH. However, more clinical and basic studies are needed to validate these hypotheses.

## Conclusions

Together, these results suggest that PFKFB3 inhibition can suppress IH angiogenesis and induce apoptosis and that targeting PFKFB3 may be a novel therapeutic strategy for IH.

## Supplementary Information

**Additional file 1: Table S1.** Clinical features of eight patients with infantile hemangioma.

**Additional file 2: Figure S1.** Hematoxylin–eosin and glucose transporter-1 staining of IH samples.

**Additional file 3: Figure S2.** Overview of the transcriptional analysis after PFKFB3 inhibition.

**Additional file 4: Table S2.** Differentially expressed genes between proliferating IH and involuting IH.

**Additional file 5: Table S3.** Expression profiles of 14 selected metabolites.

## Data Availability

All data analyzed in the present study are available from the corresponding author upon reasonable request.

## Abbreviations

IH: Infantile hemangioma
HemECs: Hemangioma-derived endothelial cells
PFKFB3: 6-Phosphofructo-2-kinase/fructose-2,6-bisphosphatase 3
DEGs: Differentially expressed genes
VEGF: Vascular endothelial growth factor
EC: Endothelial cell
PFK-1: Phosphofructokinase-1
TEM: Transmission electron microscopy
ROS: Reactive oxygen species
MVD: Microvessel density
OXPHOS: Oxidative phosphorylation
