# Supplementary material for: Blockage of glycolysis by targeting PFKFB3 suppresses the development of infantile hemangioma
Source: J Transl Med. 2023 Feb 6;21:85. doi: 10.1186/s12967-023-03932-y (PMC9901151; doi:10.1186/s12967-023-03932-y)
Supplement: Supplementary file 1 — Additional file 1: Table S1. Clinical features of eight patients with infantile hemangioma. [file 12967_2023_3932_MOESM1_ESM.docx]

Table S1. Clinical features of eight patients with infantile hemangioma.

| **Rank** | **Gender** | **Age** | **Location** | **Growth Phase** |
| --- | --- | --- | --- | --- |
| 1 | Male | 2M 25D | Elbow | Proliferating |
| 2 | Female | 6M 7D | Abdominal wall | Proliferating |
| 3 | Female | 6M 23D | Neck | Proliferating |
| 4 | Male | 1Y | Back | Involuting |
| 5 | Male | 1Y | Thoracic wall | Involuting |
| 6 | Female | 9M 20D | Thoracic wall | Involuting |
| 7 | Female | 8M 21D | Tempus | Involuting |
| 8 | Female | 2Y 5M | Neck | Involuting |
