# Supplementary figures and images for: Blockage of glycolysis by targeting PFKFB3 suppresses the development of infantile hemangioma
Source: J Transl Med. 2023 Feb 6;21:85. doi: 10.1186/s12967-023-03932-y (PMC9901151; doi:10.1186/s12967-023-03932-y)

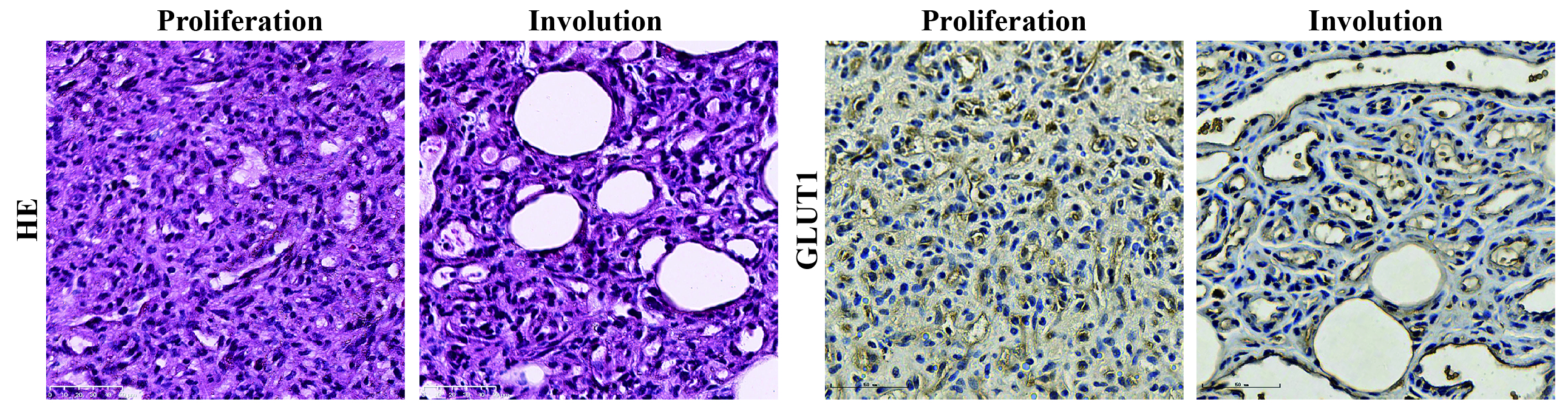

Supplement: Supplementary file 2 — Additional file 2: Figure S1. Hematoxylin–eosin and glucose transporter-1 staining of IH samples. [file 12967_2023_3932_MOESM2_ESM.jpg]

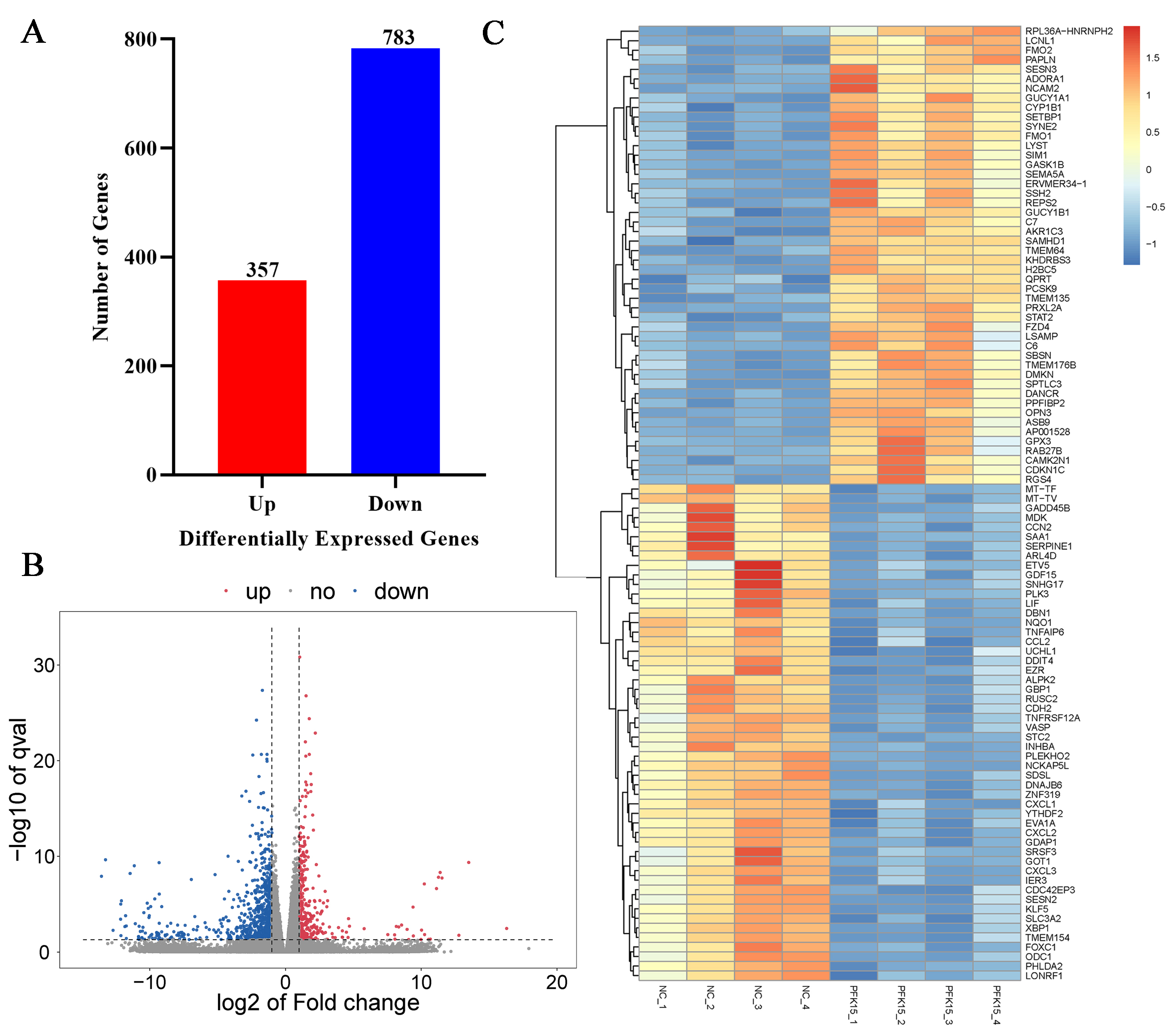

Supplement: Supplementary file 3 — Additional file 3: Figure S2. Overview of the transcriptional analysis after PFKFB3 inhibition. [file 12967_2023_3932_MOESM3_ESM.jpg]
