# Supplementary material for: Blockage of glycolysis by targeting PFKFB3 suppresses the development of infantile hemangioma
Source: J Transl Med. 2023 Feb 6;21:85. doi: 10.1186/s12967-023-03932-y (PMC9901151; doi:10.1186/s12967-023-03932-y)
Supplement: Supplementary file 4 — Additional file 4: Table S2. Differentially expressed genes between proliferating IH and involuting IH. [file 12967_2023_3932_MOESM4_ESM.docx]

| **Table S2. Differentially expressed genes between proliferating IH and involuting IH** | | | | | | | |
| --- | --- | --- | --- | --- | --- | --- | --- |
| **Rank** | **Gene Symbol** | **Mean Level of Proliferating Group** | **Mean Level of Involuting Group** | **Gene Feature** | **P-value** | **Fold Change** | **q-value** |
| 1 | LIPE | 8.004956 | 6.224793 | up | 5.40E-05 | 3.43465 | 0 |
| 2 | PFKFB3 | 7.683175 | 6.267078 | up | 5.40E-05 | 2.668626 | 0 |
| 3 | PF4 | 6.475181 | 5.702937 | up | 5.80E-05 | 1.707925 | 0 |
| 4 | TUSC5 | 7.481453 | 5.80677 | up | 6.50E-05 | 3.192492 | 0 |
| 5 | PPBP | 5.009625 | 3.693021 | up | 6.80E-05 | 2.490791 | 0 |
| 6 | AGPAT2 | 7.702292 | 6.747196 | up | 7.20E-05 | 1.93871 | 0 |
| 7 | PLIN1 | 9.397909 | 6.639229 | up | 7.90E-05 | 6.767769 | 0 |
| 8 | SIK2 | 7.954327 | 7.104464 | up | 8.10E-05 | 1.802329 | 0 |
| 9 | CIDEC | 8.341844 | 6.099295 | up | 8.20E-05 | 4.732327 | 0 |
| 10 | CALB2 | 5.560535 | 4.343878 | up | 9.60E-05 | 2.324075 | 0 |
| 11 | TM7SF2 | 6.575558 | 5.894596 | up | 9.80E-05 | 1.603209 | 0 |
| 12 | GYG2 | 7.325422 | 5.950724 | up | 9.90E-05 | 2.593136 | 0 |
| 13 | DGAT2 | 8.629005 | 5.851535 | up | 0.000106 | 6.856491 | 0 |
| 14 | RBP4 | 7.58854 | 6.044471 | up | 0.000133 | 2.916157 | 0 |
| 15 | NNAT | 7.747798 | 5.740875 | up | 0.000137 | 4.019239 | 0 |
| 16 | DNM1L | 7.002451 | 7.636685 | down | 0.000147 | -1.55211 | 0.015268 |
| 17 | ACO1 | 7.504804 | 6.726673 | up | 0.000178 | 1.714908 | 0 |
| 18 | KLB | 5.183965 | 3.810961 | up | 0.000203 | 2.590093 | 0 |
| 19 | LGALS12 | 5.930963 | 4.93457 | up | 0.000207 | 1.995005 | 0 |
| 20 | MARC1 | 6.372828 | 5.359991 | up | 0.000213 | 2.017875 | 0 |
| 21 | OR6Q1 | 4.07316 | 3.464273 | up | 0.000223 | 1.525081 | 0 |
| 22 | TPMT | 6.069076 | 6.81718 | down | 0.000226 | -1.67958 | 0.015268 |
| 23 | PLIN4 | 8.607193 | 6.589523 | up | 0.000234 | 4.049292 | 0 |
| 24 | SLC19A3 | 6.344323 | 4.841517 | up | 0.000242 | 2.833934 | 0 |
| 25 | C10orf10 | 7.420604 | 6.157776 | up | 0.000249 | 2.399655 | 0 |
| 26 | RPS6KA3 | 7.186773 | 7.996339 | down | 0.000264 | -1.75268 | 0.015268 |
| 27 | PC | 6.054378 | 5.45272 | up | 0.000282 | 1.517459 | 0 |
| 28 | HEPACAM | 5.070812 | 4.468587 | up | 0.000318 | 1.518056 | 0 |
| 29 | CIDEA | 6.2312 | 5.539669 | up | 0.000354 | 1.614997 | 0 |
| 30 | TKT | 7.490282 | 6.685009 | up | 0.000368 | 1.747476 | 0 |
| 31 | MGST1 | 5.736524 | 4.367813 | up | 0.00039 | 2.582398 | 0 |
| 32 | SCD | 10.033451 | 7.43518 | up | 4.00E-04 | 6.055605 | 0 |
| 33 | EPB41L4B | 5.316123 | 4.471608 | up | 0.000403 | 1.795661 | 0 |
| 34 | SAA1 | 5.601082 | 4.743646 | up | 0.000429 | 1.811816 | 0 |
| 35 | SLIRP | 8.19167 | 8.96285 | down | 0.000442 | -1.70667 | 0.019373 |
| 36 | RINT1 | 5.510065 | 6.103196 | down | 0.000452 | -1.50852 | 0.019373 |
| 37 | GPAM | 8.693587 | 6.238877 | up | 0.000488 | 5.482032 | 0 |
| 38 | PCK1 | 5.414712 | 4.735093 | up | 0.000506 | 1.601717 | 0 |
| 39 | FASN | 8.194719 | 6.319832 | up | 0.000508 | 3.667729 | 0 |
| 40 | THRSP | 9.575095 | 6.351122 | up | 0.000511 | 9.343562 | 0 |
| 41 | TF | 5.448055 | 4.622139 | up | 0.000522 | 1.77266 | 0 |
| 42 | DPH6 | 5.06578 | 5.812449 | down | 0.000538 | -1.67791 | 0.019373 |
| 43 | TMEM37 | 6.880119 | 6.128027 | up | 0.000555 | 1.684233 | 0 |
| 44 | SVIP | 5.348242 | 6.211428 | down | 0.000566 | -1.81905 | 0.019373 |
| 45 | HCAR2 | 5.502427 | 4.813349 | up | 0.000633 | 1.612253 | 0.005254 |
| 46 | TMEM132C | 6.323451 | 5.615349 | up | 0.000719 | 1.633654 | 0.005254 |
| 47 | ASUN | 5.836689 | 6.491834 | down | 0.000726 | -1.57477 | 0.019373 |
| 48 | ALDOC | 5.825296 | 4.944149 | up | 0.000731 | 1.84184 | 0.005254 |
| 49 | LOC389831 | 5.179659 | 6.259404 | down | 0.000768 | -2.11366 | 0.021184 |
| 50 | KCNIP2 | 6.212429 | 5.39376 | up | 0.000775 | 1.763778 | 0.005254 |
| 51 | AZGP1 | 6.043856 | 5.443343 | up | 0.000792 | 1.516256 | 0.005254 |
| 52 | HRASLS5 | 6.335157 | 5.225619 | up | 0.000805 | 2.157765 | 0.005254 |
| 53 | ACACB | 8.54989 | 6.991155 | up | 0.000879 | 2.945953 | 0.005254 |
| 54 | S100B | 6.443918 | 5.809087 | up | 0.000918 | 1.552757 | 0.005254 |
| 55 | RBP7 | 6.763764 | 5.992071 | up | 0.001111 | 1.707273 | 0.005254 |
| 56 | APBB1IP | 5.817522 | 5.164077 | up | 0.001122 | 1.572919 | 0.005254 |
| 57 | G0S2 | 8.919931 | 7.757394 | up | 0.001221 | 2.238507 | 0.006075 |
| 58 | CHRM4 | 5.234774 | 4.525272 | up | 0.001261 | 1.635239 | 0.006075 |
| 59 | RARRES2 | 7.770037 | 6.954088 | up | 0.001285 | 1.760456 | 0.006075 |
| 60 | TCAIM | 5.52236 | 6.462989 | down | 0.001312 | -1.91937 | 0.027907 |
| 61 | UBR1 | 7.021902 | 7.626192 | down | 0.001327 | -1.52023 | 0.027907 |
| 62 | ADIPOQ | 9.634881 | 6.324283 | up | 0.001354 | 9.921774 | 0.006075 |
| 63 | MSTN | 4.002581 | 5.722794 | down | 0.001387 | -3.29485 | 0.027907 |
| 64 | AIMP1 | 6.646171 | 7.408011 | down | 0.001439 | -1.69565 | 0.027907 |
| 65 | SLC25A1 | 7.386942 | 6.767586 | up | 0.001448 | 1.53619 | 0.006075 |
| 66 | BRCC3 | 6.40623 | 7.065481 | down | 0.001472 | -1.57926 | 0.027907 |
| 67 | BOK | 7.172717 | 6.5845 | up | 0.00154 | 1.503387 | 0.006075 |
| 68 | MAPK6 | 7.100932 | 7.786781 | down | 0.001613 | -1.60865 | 0.027907 |
| 69 | SCRN3 | 5.903408 | 6.714637 | down | 0.001658 | -1.75471 | 0.027907 |
| 70 | DHRS3 | 7.158675 | 6.342976 | up | 0.001704 | 1.760151 | 0.006075 |
| 71 | FAH | 5.943248 | 5.192398 | up | 0.00182 | 1.682784 | 0.006075 |
| 72 | RBM41 | 5.73849 | 6.367634 | down | 0.001827 | -1.54665 | 0.027907 |
| 73 | AACS | 6.388677 | 5.494536 | up | 0.001994 | 1.858502 | 0.006075 |
| 74 | IFIT1 | 4.557814 | 5.221481 | down | 0.00203 | -1.5841 | 0.048872 |
| 75 | APOB | 4.248909 | 3.65754 | up | 0.002103 | 1.506676 | 0.006075 |
| 76 | ACLY | 8.786057 | 7.558633 | up | 0.002116 | 2.341485 | 0.006075 |
| 77 | PNPLA2 | 8.167093 | 6.975271 | up | 0.002143 | 2.28441 | 0.006075 |
| 78 | DGAT1 | 7.174825 | 6.549213 | up | 0.002229 | 1.542865 | 0.006075 |
| 79 | AADAC | 4.478656 | 3.297306 | up | 0.00225 | 2.267888 | 0.006075 |
| 80 | CLMP | 7.382894 | 6.453089 | up | 0.002263 | 1.905018 | 0.006075 |
| 81 | HSDL1 | 6.343147 | 6.931478 | down | 0.002287 | -1.50351 | 0.048872 |
| 82 | GLUL | 7.994275 | 6.989638 | up | 0.00237 | 2.006439 | 0.01113 |
| 83 | PHGDH | 6.286507 | 5.620299 | up | 0.002387 | 1.586897 | 0.01113 |
| 84 | CCNG1 | 7.382502 | 8.210142 | down | 0.002404 | -1.77478 | 0.048872 |
| 85 | BCAS2 | 6.989991 | 7.657343 | down | 0.002414 | -1.58815 | 0.048872 |
| 86 | SFRP1 | 7.111841 | 6.450714 | up | 0.002451 | 1.581318 | 0.01113 |
| 87 | LPL | 8.90187 | 7.387141 | up | 0.002564 | 2.857451 | 0.01113 |
| 88 | RETSAT | 7.260707 | 6.443996 | up | 0.002587 | 1.761386 | 0.01113 |
| 89 | GALNT1 | 7.3203 | 7.994863 | down | 0.002859 | -1.59611 | 0.048872 |
| 90 | C12orf4 | 6.908818 | 7.623467 | down | 0.002887 | -1.64108 | 0.048872 |
| 91 | CLU | 7.802604 | 6.986153 | up | 0.003119 | 1.761068 | 0.01113 |
| 92 | GIN1 | 4.103966 | 4.88304 | down | 0.003171 | -1.71603 | 0.080832 |
| 93 | ERGIC2 | 7.836719 | 8.636808 | down | 0.003216 | -1.74121 | 0.080832 |
| 94 | DZIP3 | 4.896872 | 5.666431 | down | 0.003231 | -1.70475 | 0.080832 |
| 95 | HMGCLL1 | 4.576915 | 5.483177 | down | 0.00333 | -1.87418 | 0.080832 |
| 96 | INHBB | 5.826984 | 4.689233 | up | 0.003339 | 2.200377 | 0.01113 |
| 97 | UFL1 | 7.273203 | 8.004245 | down | 0.003366 | -1.65984 | 0.080832 |
| 98 | ANKRD26 | 4.727115 | 5.346181 | down | 0.003421 | -1.53588 | 0.080832 |
| 99 | PSMC2 | 8.266649 | 8.896053 | down | 0.003538 | -1.54693 | 0.080832 |
| 100 | CENPQ | 4.084645 | 4.813025 | down | 0.003606 | -1.65678 | 0.080832 |
| 101 | C12orf29 | 5.923272 | 6.645166 | down | 0.003836 | -1.64935 | 0.080832 |
| 102 | LEP | 6.337876 | 4.690717 | up | 0.003952 | 3.132161 | 0.015268 |
| 103 | CTNNA3 | 4.446618 | 5.511019 | down | 0.003992 | -2.0913 | 0.080832 |
| 104 | PYGL | 7.426481 | 6.447458 | up | 0.004141 | 1.971131 | 0.015268 |
| 105 | TNFRSF21 | 7.982146 | 7.284708 | up | 0.004272 | 1.621623 | 0.015268 |
| 106 | GALR3 | 6.248024 | 5.639346 | up | 0.004323 | 1.524862 | 0.015268 |
| 107 | NEDD1 | 7.13869 | 8.198716 | down | 0.004364 | -2.08497 | 0.080832 |
| 108 | NEDD4 | 6.532702 | 7.173191 | down | 0.004402 | -1.55886 | 0.080832 |
| 109 | PTPRF | 7.174019 | 6.448842 | up | 0.00451 | 1.653103 | 0.015268 |
| 110 | COPS4 | 5.396931 | 6.019929 | down | 0.005086 | -1.54007 | 0.135284 |
| 111 | APOOL | 6.161332 | 6.751525 | down | 0.005162 | -1.50545 | 0.135284 |
| 112 | NT5C3A | 4.681869 | 5.471366 | down | 0.005559 | -1.72847 | 0.135284 |
| 113 | RICTOR | 7.024439 | 7.627829 | down | 0.005622 | -1.51928 | 0.135284 |
| 114 | RPAP3 | 6.065869 | 6.846178 | down | 0.005714 | -1.7175 | 0.135284 |
| 115 | ATAD1 | 6.886186 | 7.630323 | down | 0.005743 | -1.67497 | 0.135284 |
| 116 | GRN | 8.847001 | 8.209121 | up | 0.005888 | 1.556042 | 0.019373 |
| 117 | DPH3 | 5.915296 | 6.518501 | down | 0.006155 | -1.51909 | 0.135284 |
| 118 | KCTD9 | 6.249939 | 6.838685 | down | 0.006554 | -1.50394 | 0.135284 |
| 119 | ENPP4 | 5.02066 | 5.767102 | down | 0.006596 | -1.67765 | 0.135284 |
| 120 | ACVR1C | 6.737856 | 5.316207 | up | 0.006616 | 2.678915 | 0.019373 |
| 121 | TRMT13 | 4.513191 | 5.151766 | down | 0.006718 | -1.55679 | 0.135284 |
| 122 | NDUFB6 | 4.833278 | 5.61309 | down | 0.006905 | -1.71691 | 0.135284 |
| 123 | SOD3 | 6.26814 | 5.611089 | up | 0.006927 | 1.576856 | 0.019373 |
| 124 | MOSPD1 | 6.21652 | 6.91903 | down | 0.006988 | -1.62733 | 0.135284 |
| 125 | PSMA3 | 6.611358 | 7.341515 | down | 0.007026 | -1.65882 | 0.135284 |
| 126 | USP15 | 6.540176 | 7.308776 | down | 0.007035 | -1.70362 | 0.135284 |
| 127 | PYROXD1 | 5.768664 | 6.623583 | down | 0.007073 | -1.80866 | 0.135284 |
| 128 | LYRM7 | 5.987886 | 6.635411 | down | 0.007202 | -1.56648 | 0.135284 |
| 129 | FOSB | 7.030425 | 6.098253 | up | 0.007236 | 1.908147 | 0.019373 |
| 130 | ZBTB18 | 5.628573 | 6.432506 | down | 0.00729 | -1.74585 | 0.135284 |
| 131 | ZNF24 | 6.884576 | 7.584008 | down | 0.007321 | -1.62387 | 0.135284 |
| 132 | PM20D2 | 4.936669 | 5.945001 | down | 0.007337 | -2.01158 | 0.135284 |
| 133 | RASSF8 | 5.642144 | 6.228688 | down | 0.007433 | -1.50165 | 0.17509 |
| 134 | AP1S2 | 5.685044 | 6.408531 | down | 0.007755 | -1.65117 | 0.17509 |
| 135 | CNOT7 | 6.522265 | 7.115584 | down | 0.007772 | -1.50871 | 0.17509 |
| 136 | ADH1A | 5.635653 | 4.636554 | up | 0.007965 | 1.998751 | 0.019373 |
| 137 | LTN1 | 6.113261 | 6.737056 | down | 0.007993 | -1.54092 | 0.17509 |
| 138 | VTA1 | 7.520066 | 8.135627 | down | 0.008089 | -1.53215 | 0.17509 |
| 139 | PSMD12 | 6.637765 | 7.346278 | down | 0.008111 | -1.63412 | 0.17509 |
| 140 | ADH1B | 9.804235 | 7.357809 | up | 0.008197 | 5.450639 | 0.019373 |
| 141 | HBS1L | 6.1742 | 6.858784 | down | 0.008405 | -1.60724 | 0.17509 |
| 142 | NIPSNAP3B | 4.845031 | 5.959644 | down | 0.008459 | -2.16537 | 0.17509 |
| 143 | MMP9 | 6.575098 | 5.938179 | up | 0.008477 | 1.555005 | 0.019373 |
| 144 | LRP1 | 9.208243 | 8.563738 | up | 0.008508 | 1.563203 | 0.021184 |
| 145 | RBL1 | 5.57347 | 6.216811 | down | 0.008518 | -1.56194 | 0.17509 |
| 146 | CMAS | 6.358758 | 7.052671 | down | 0.008544 | -1.61767 | 0.17509 |
| 147 | TNMD | 5.099576 | 6.324679 | down | 0.008616 | -2.33772 | 0.17509 |
| 148 | CUL5 | 6.824302 | 7.659208 | down | 0.008775 | -1.78374 | 0.17509 |
| 149 | GLYAT | 4.809453 | 3.547912 | up | 0.008795 | 2.397517 | 0.021184 |
| 150 | RRM2B | 6.133245 | 6.951175 | down | 0.008803 | -1.76288 | 0.17509 |
| 151 | NUDT12 | 4.399066 | 5.229995 | down | 0.008815 | -1.77883 | 0.17509 |
| 152 | MMD | 7.5293 | 6.39451 | up | 0.008889 | 2.195867 | 0.021184 |
| 153 | MYNN | 6.124028 | 6.780149 | down | 0.008931 | -1.57584 | 0.17509 |
| 154 | FKBP5 | 7.748083 | 6.9424 | up | 0.008955 | 1.747973 | 0.021184 |
| 155 | ASNSD1 | 6.628619 | 7.329128 | down | 0.008981 | -1.62508 | 0.17509 |
| 156 | CPSF2 | 6.586957 | 7.195011 | down | 0.009439 | -1.5242 | 0.17509 |
| 157 | LMBRD2 | 5.685568 | 6.337832 | down | 0.009474 | -1.57163 | 0.17509 |
| 158 | COL1A2 | 10.772319 | 10.069907 | up | 0.009676 | 1.627223 | 0.021184 |
| 159 | NDUFAF4 | 4.759228 | 5.424358 | down | 0.009744 | -1.58571 | 0.17509 |
| 160 | KCNK5 | 5.015247 | 4.395568 | up | 0.009953 | 1.536533 | 0.021184 |
| 161 | COX20 | 6.956711 | 7.602675 | down | 0.010285 | -1.56478 | 0.17509 |
| 162 | CNTFR | 6.874032 | 6.280524 | up | 0.010419 | 1.508911 | 0.021184 |
| 163 | ASS1 | 7.598793 | 6.493718 | up | 0.010927 | 2.1511 | 0.021184 |
| 164 | RB1CC1 | 6.547205 | 7.234772 | down | 0.011036 | -1.61057 | 0.17509 |
| 165 | VAMP4 | 6.085396 | 6.85672 | down | 0.01108 | -1.70684 | 0.17509 |
| 166 | PTPRS | 7.443193 | 6.848212 | up | 0.011094 | 1.510452 | 0.021184 |
| 167 | TMEM38B | 6.095225 | 7.404834 | down | 0.011286 | -2.47874 | 0.17509 |
| 168 | BLOC1S2 | 5.731381 | 6.360123 | down | 0.011286 | -1.54622 | 0.17509 |
| 169 | ECE1 | 8.448895 | 7.810234 | up | 0.011384 | 1.556883 | 0.021184 |
| 170 | FOPNL | 6.92699 | 7.637334 | down | 0.011385 | -1.63619 | 0.17509 |
| 171 | ALDH2 | 8.419798 | 7.424383 | up | 0.011461 | 1.993654 | 0.021184 |
| 172 | CASD1 | 6.411108 | 7.048488 | down | 0.011686 | -1.5555 | 0.17509 |
| 173 | USP47 | 7.581414 | 8.202921 | down | 0.011694 | -1.53848 | 0.17509 |
| 174 | ADAM12 | 7.036429 | 6.301128 | up | 0.011978 | 1.664744 | 0.021184 |
| 175 | PDE3B | 6.106626 | 4.648519 | up | 0.012257 | 2.747475 | 0.021184 |
| 176 | LGALS3BP | 8.420374 | 7.792639 | up | 0.012505 | 1.545137 | 0.021184 |
| 177 | OLFM2 | 7.725815 | 6.966568 | up | 0.012984 | 1.692607 | 0.021184 |
| 178 | SPON1 | 7.031588 | 6.37496 | up | 0.013136 | 1.576393 | 0.021184 |
| 179 | COL3A1 | 10.89711 | 10.214092 | up | 0.013183 | 1.605494 | 0.021184 |
| 180 | VWF | 8.529173 | 7.89452 | up | 0.013518 | 1.552565 | 0.021184 |
| 181 | COL6A2 | 8.84511 | 8.205056 | up | 0.014196 | 1.558388 | 0.021184 |
| 182 | DSG2 | 4.165287 | 3.242468 | up | 0.015396 | 1.895816 | 0.027907 |
| 183 | FOS | 8.116959 | 7.17754 | up | 0.015634 | 1.917756 | 0.027907 |
| 184 | LAMB1 | 8.780807 | 8.087507 | up | 0.01572 | 1.616978 | 0.027907 |
| 185 | ANG | 6.846706 | 6.235589 | up | 0.017233 | 1.527441 | 0.027907 |
| 186 | COL1A1 | 10.172257 | 9.335543 | up | 0.01726 | 1.785977 | 0.027907 |
| 187 | CPM | 6.991485 | 6.055786 | up | 0.017324 | 1.912817 | 0.027907 |
| 188 | NOTCH1 | 7.69112 | 7.096966 | up | 0.018033 | 1.509587 | 0.027907 |
| 189 | GPD1 | 7.421094 | 6.269558 | up | 0.018825 | 2.221503 | 0.027907 |
| 190 | MGLL | 6.847439 | 6.257901 | up | 0.018884 | 1.504765 | 0.027907 |
| 191 | ELN | 8.316755 | 7.491594 | up | 0.020003 | 1.771732 | 0.027907 |
| 192 | ACSL1 | 9.084284 | 7.297253 | up | 0.020109 | 3.45104 | 0.027907 |
| 193 | CCL3L3 | 5.794793 | 5.012804 | up | 0.020337 | 1.7195 | 0.027907 |
| 194 | CCL3L3 | 6.182365 | 5.452929 | up | 0.022196 | 1.65799 | 0.027907 |
| 195 | KLF2 | 8.012131 | 7.362205 | up | 0.022256 | 1.569087 | 0.027907 |
| 196 | OLFML2B | 8.7471 | 8.143735 | up | 0.022347 | 1.519256 | 0.027907 |
| 197 | PLA2G16 | 8.231093 | 7.259317 | up | 0.023613 | 1.961254 | 0.027907 |
| 198 | FLG2 | 2.652588 | 1.993204 | up | 0.025808 | 1.579409 | 0.027907 |
| 199 | ECHS1 | 7.866361 | 7.252949 | up | 0.026244 | 1.529873 | 0.027907 |
| 200 | MMP14 | 8.609122 | 7.927989 | up | 0.026278 | 1.603398 | 0.027907 |
| 201 | EGR1 | 8.921863 | 8.114745 | up | 0.026988 | 1.749713 | 0.048872 |
| 202 | PI16 | 7.711712 | 7.055811 | up | 0.027404 | 1.5756 | 0.048872 |
| 203 | CDO1 | 6.926015 | 6.303188 | up | 0.027728 | 1.53989 | 0.048872 |
| 204 | DSP | 5.09888 | 4.196517 | up | 0.027785 | 1.869125 | 0.048872 |
| 205 | PLVAP | 9.603116 | 8.584076 | up | 0.029348 | 2.026571 | 0.048872 |
| 206 | PPAP2B | 8.854494 | 8.239859 | up | 0.02958 | 1.53117 | 0.048872 |
| 207 | HOXD10 | 5.285013 | 4.685663 | up | 0.031217 | 1.515034 | 0.048872 |
| 208 | ACAN | 6.727965 | 6.084691 | up | 0.031347 | 1.56187 | 0.048872 |
| 209 | MYO1C | 8.733598 | 8.122359 | up | 0.031543 | 1.52757 | 0.048872 |
| 210 | IRS2 | 6.489814 | 5.700261 | up | 0.032115 | 1.728539 | 0.048872 |
| 211 | DDIT4 | 7.665187 | 6.872596 | up | 0.032723 | 1.732183 | 0.048872 |
| 212 | COLGALT1 | 8.614439 | 8.028935 | up | 0.032938 | 1.500563 | 0.048872 |
| 213 | PDE2A | 6.712894 | 6.026902 | up | 0.034017 | 1.608807 | 0.048872 |
| 214 | GPC3 | 7.300375 | 6.51116 | up | 0.035266 | 1.728134 | 0.048872 |
| 215 | CCL21 | 7.863298 | 6.201488 | up | 0.035488 | 3.164134 | 0.048872 |
| 216 | S1PR3 | 7.412207 | 6.484624 | up | 0.037477 | 1.902087 | 0.048872 |
| 217 | CCL3 | 5.931726 | 5.116862 | up | 0.038645 | 1.759132 | 0.048872 |
| 218 | SLCO2A1 | 7.115966 | 6.357119 | up | 0.039186 | 1.692138 | 0.048872 |
| 219 | PLA2G2A | 6.263928 | 5.655401 | up | 0.041136 | 1.524702 | 0.048872 |
| 220 | HSPG2 | 8.659994 | 8.064907 | up | 0.045764 | 1.510564 | 0.048872 |
| 221 | HTRA3 | 7.53738 | 6.781097 | up | 0.04648 | 1.689133 | 0.048872 |
| 222 | ZFP36 | 8.648366 | 7.947631 | up | 0.048394 | 1.625332 | 0.048872 |
