# Supplementary material for: Blockage of glycolysis by targeting PFKFB3 suppresses the development of infantile hemangioma
Source: J Transl Med. 2023 Feb 6;21:85. doi: 10.1186/s12967-023-03932-y (PMC9901151; doi:10.1186/s12967-023-03932-y)
Supplement: Supplementary file 5 — Additional file 5: Table S3. Expression profiles of 14 selected metabolites. [file 12967_2023_3932_MOESM5_ESM.docx]

| **Rank** | **Compound name** | **Mean of PFK15 group** | **Mean of Control group** | **VIP** | **P-VALUE** | **Q-VALUE** | **FOLD CHANGE** | **LOG_FOLDCHANGE** |
| --- | --- | --- | --- | --- | --- | --- | --- | --- |
| 1 | Cyclic 3',5'-AMP | 6.127285945 | 4.329491133 | 1.293107505 | 5.5912E-05 | 0.00251604 | 1.415243907 | 0.501050712 |
| 2 | 3-Phosphoglyceric acid | 871.4383645 | 294.6401209 | 1.437805818 | 0.000664169 | 0.01494381 | 2.957636461 | 1.564444734 |
| 3 | Phosphoenolpyruvic acid | 430.0959551 | 126.0228879 | 1.439446836 | 0.001479988 | 0.01708747 | 3.412840018 | 1.770972788 |
| 4 | Fumaric acid | 887.6833916 | 321.4006968 | 1.442350063 | 0.001518886 | 0.01708747 | 2.761921179 | 1.465672148 |
| 5 | Malic acid | 5914.79284 | 1985.578311 | 1.4533558 | 0.002143943 | 0.01929549 | 2.978876636 | 1.574768378 |
| 6 | Cysteic acid | 256.1276468 | 93.04791162 | 1.147470934 | 0.005297009 | 0.03411886 | 2.752642616 | 1.460817312 |
| 7 | Glycerol 3-phosphate | 54.61688162 | 42.25688295 | 1.184862892 | 0.005307378 | 0.03411886 | 1.292496696 | 0.370160593 |
| 8 | Glyceric acid | 157.2929535 | 64.37045415 | 1.196773878 | 0.006469039 | 0.03461938 | 2.443558237 | 1.288983489 |
| 9 | L-Lactic acid | 83325.23663 | 21184.08515 | 1.319510223 | 0.006923876 | 0.03461938 | 3.933388486 | 1.975772683 |
| 10 | Hippuric acid | 89.83484258 | 38.16217412 | 1.29423531 | 0.008658345 | 0.03708781 | 2.354028423 | 1.23513174 |
| 11 | 4-Hydroxyphenylpyruvic acid | 85.18398146 | 38.20530978 | 1.203653817 | 0.009254503 | 0.03708781 | 2.22963724 | 1.156809004 |
| 12 | Fructose 1,6-bisphosphate | 355.5217298 | 72.80854935 | 1.364209328 | 0.009890083 | 0.03708781 | 4.882966808 | 2.287757971 |
| 13 | Gluconic acid | 194.6080644 | 87.10813987 | 1.281911521 | 0.013273391 | 0.04266447 | 2.234097349 | 1.159692052 |
| 14 | Sedoheptulose 7-phosphate | 1359.061064 | 226.3130646 | 1.393539054 | 0.016494784 | 0.04948435 | 6.005225842 | 2.586218503 |

**Table S3. Expression profiles of 14 selected metabolites**
